# Physiological Characteristics and Comparative Secretome Analysis of *Morchella importuna* Grown on Glucose, Rice Straw, Sawdust, Wheat Grain, and MIX Substrates

**DOI:** 10.3389/fmicb.2021.636344

**Published:** 2021-05-25

**Authors:** YingLi Cai, XiaoLong Ma, QianQian Zhang, FuQiang Yu, Qi Zhao, Wei Huang, JiaXin Song, Wei Liu

**Affiliations:** ^1^Institute of Vegetable, Wuhan Academy of Agricultural Sciences, Wuhan, China; ^2^Institute of Applied Mycology, Huazhong Agricultural University, Wuhan, China; ^3^Germplasm Bank of Wild Species in Southwestern China, Yunnan Key Laboratory for Fungal Diversity and Green Development, Kunming Institute of Botany, Chinese Academy of Sciences, Kunming, China; ^4^Institute of Applied Mycology, Southwest Forestry University, Kunming, China

**Keywords:** secretory proteome, enzyme activity, substrate utilization, amylase, nutritional metabolism, CAZYme

## Abstract

Morels (*Morchella* sp.) are economically important edible macro-fungi, which can grow on various synthetic or semi-synthetic media. However, the complex nutritional metabolism and requirements of these fungi remain ill-defined. This study, based on the plant biomass commonly used in the artificial cultivation of morels, assessed and compared the growth characteristics and extracellular enzymes of *Morchella importuna* cultivated on glucose, rice straw, sawdust, wheat grain, and a mixture of equal proportions of the three latter plant substrates (MIX). *M. importuna* could grow on all five tested media but displayed significant variations in mycelial growth rate, biomass, and sclerotium yield on the different media. The most suitable medium for *M. importuna* was wheat and wheat-containing medium, followed by glucose, while rice straw and sawdust were the least suitable. A total of 268 secretory proteins were identified by liquid chromatography coupled with tandem mass spectrometry detection. Functional classification and label-free comparative analysis of these proteins revealed that carbohydrate-active enzyme (CAZYme) proteins were the predominant component of the secretome of *M. importuna*, followed by protease, peptidase, and other proteins. The abundances of CAZYme proteins differed among the tested media, ranging from 64% on glucose to 88% on rice straw. The CAZYme classes of glycoside hydrolases and carbohydrate-binding module were enriched in the five secretomes. Furthermore, the enzyme activities of CMCase, lignase, amylase, xylase, pNPCase, and pNPGase were detected during the continuous culture of *M. importuna* in MIX medium, and the relative expression of the corresponding genes were detected by quantitative real-time PCR. The combined data of growth potential, secretome, extracellular enzyme activity, and gene expression on different substrates inferred that *M. importuna* was weak in lignocellulose degradation but a good starch decomposer. Specifically, in terms of the degradation of cellulose, the ability to degrade cellulose into oligosaccharides was weaker compared with further degradation into monosaccharides, and this might be the speed-limiting step of cellulose utilization in *M. importuna*. In addition, *M. importuna* had a strong ability to decompose various hemicellulose glycosidic bonds, especially α- and β-galactosidase. Only a very few lignin-degradation-related proteins were detected, and these were in low abundance, consistent with the presence of weak lignin degradation ability. Furthermore, the presence of lipase and chitinase implied that *M. importuna* was capable of decomposition of its own mycelia *in vitro*. The study provides key data that facilitates a further understanding of the complex nutritional metabolism of *M. importuna*.

## Introduction

The secretome represents all the proteins and cellular machineries that are secreted outside the plasma membrane into the environment or extracellular matrix by a cell (Tjalsma et al., 2000; McCotter et al., 2016). Microorganisms such as bacteria and fungi produce unique substrate-specific secretomes under different environmental conditions. In fungi, secretomic studies are expected to facilitate an understanding of the extracellular enzymatic mechanisms involved in the degradation of plant tissues and complex materials in the environment, which are subsequently utilized by hypha to grow and elongate (Bouws et al., 2008; Girard et al., 2013). In the past decade, advances in protein identification techniques and genome sequencing have enabled a detailed investigation of the secretomes of many saprophytic, pathogenic, and symbiotic fungal species, revealing rich, diverse, and highly specific enzymatic profiles (Shah et al., 2009; Jun et al., 2011; Liu et al., 2013; Sista Kameshwar and Qin, 2018). Research on substrate degradation has predominantly focused on various saprophytic fungi of Basidiomycetes, such as white rot, brown rot, and soft rot fungi (Fernández-Fueyo et al., 2016; Cai et al., 2017). These fungi have different degradation abilities for plant organic matter and play important roles in the ecological cycle and chemical industry and as food and medicine. Secretomes of some plant pathogens, mycorrhizal symbiotic fungi, and some micro-ascomycetes, such as *Aspergillus*, *Trichoderma* sp., *Penicillium* sp., *Botrytis* sp., *etc*. have also been systematically studied (Shah et al., 2009; Jun et al., 2011; Liu et al., 2013; Volke-Sepulveda et al., 2016). However, studies on macro-ascomycetes are limited. For macro-fungi, especially commercially cultivated mushrooms, agricultural residues are generally considered the best substrates for fruiting-body-producing fungi that produce an abundance of ligninolytic enzymes to utilize various lignocellulosic residues (Fan et al., 2008). Thus, elucidating the mechanisms of enzyme secretion and utilization in these fungi is worthwhile and may have implications for substrate choice in commercial cultivation.

True morels (*Morchella* spp.), belonging to Ascomycota, are economically important edible macro-fungi that are widely appealing due to their commercial value, beautiful appearance, medicinal properties, and unique and appetizing flavor (Liu et al., 2017). The ecological types of *Morchella* species remain controversial. *Morchella esculenta*, *Morchella elata*, and *Morchella rotunda* have a typical ectomycorrhizal structure (Buscot and Roux, 1987; Buscot and Kottke, 1990). *Morchella sextelata*, *Morchella spongiola*, and *Morchella crassipes* can be endophytic with *Bromus tectorum*, *Gymnadenia conopsea*, and maize (*Zea mays* var. *saccharate* and *Zea mays* var. *indentata*), respectively (Stark et al., 2009; Yu et al., 2016; Phanpadith et al., 2020). *Morchella rufobrunnea*, *Morchella importuna*, *M. sextelata*, and *Morchella eximia* are saprophytic species that do not require the participation of any living plants in artificial cultivation (Ower et al., 1986; He et al., 2015b; Liu et al., 2017). However, all morel species grow well on various synthetic or semisynthetic media with saprophytic growth characteristics (Liu et al., 2017). This distinctive facultative ecological type may be related to the complex substrate-degrading enzymes of these fungal species, but there is a lack of systematic research to support this theory.

Wheat (wheat, barley, and rye) was always used as a substrate during the domestication and cultivation of morels (Robbins and Hervey, 1959; Hervey et al., 1978; Ower et al., 1986). Subsequently, wheat bran, peanut shell, rice straw, sawdust, pine needles, fallen leaves, apple pomace, *etc*., were also included (Papinutti and Lechner, 2008; Kanwal and Reddy, 2011). Recent morel cultivation technology has used wheat, sawdust, and rice straw as the main materials to make spawn and exogenous nutrition bag, used for the decomposition and transformation of morel mycelium (vegetative growth), to provide sexual reproduction (fruiting) in a later stage of cultivation (Liu et al., 2017). Some enzyme activities and functional genes involved in the degradation of lignocellulosic substrates by *Morchella* species have been studied (Kanwal and Reddy, 2011; Tan et al., 2019). However, there are no systematic studies on the degradation mechanism of these substrates by the cultivable species *M. importuna*. Cellulolytic complexes of *M. conica* on cellular powder medium analysis indicated that the extracellular enzyme components comprised both endoglucanases and cellobiohydrolases, which played an important role in the practical hydrolytic processing of lignocellulosic materials (Cavazzoni and Manzoni, 1994). Gramss et al. (1998) analyzed the oxidative enzymes of *M. conica*, *M. elata*, and *M. esculenta* by using spot tests and revealed that all three species had polyphenol oxidases, laccase, tyrosinase, and peroxidase activities (Gramss et al., 1998). Guaiacol or o-dianisidine dihydrochloride can effectively induce *M. conica*, *M. elata*, and *M. spongiola* to produce significantly enhanced extracellular laccase-like multicopper oxidase activity (LMCO) (Kellner et al., 2007).

Different carbon sources induce various extracellular enzymes that affect the growth and development of fungi. On wheat bran medium, *M. esculenta* had high activities of endoglucanase, β-glucosidase, amylase, and laccase, and the change in enzyme activity corresponded to the degradation and utilization of the substrate and subsequently affected the growth and development of the fungus (Papinutti and Lechner, 2008). Maximum biomass of *M. esculenta* was obtained on wheat bran and rolled oat medium, while a large number of sclerotia were formed on wheat bran plus corn starch which contains fast metabolic carbon sources (Papinutti and Lechner, 2008). Kanwal and Reddy (2014) studied the effects of various carbon sources on sclerotium formation of *M. crassipes* and observed maximum laccase activity following the growth of the fungi on wheat grains, whereas maximum activities of manganese-dependent peroxidase and lignin peroxidase were observed when rice straw was used (Kanwal and Reddy, 2014). Under the novel morel cultivation mode, using an exogenous nutrition bag as supplement material, over 90% of amylose, 72% of amylopectin, and some lignin in the exogenous nutrition bag were metabolized during the entire growth process, while the consumption of cellulose, hemicellulose, and pectin was relatively low (Tan et al., 2019). Laccase genes play a key role in lignin degradation; one of the two laccase genes (laccase-like multicopper oxidase), MiLacA, in *M. importuna* was expressed at much higher levels throughout the entire course of artificial cultivation, and the recombinant MiLacA protein in *Pichia pastoris* showed simultaneous laccase and polyphenol-oxidase activities (Zhang et al., 2019).

The complete genome of *M. importuna* has been sequenced and contains numerous genes annotated as related to lignocellulose degradation (Liu et al., 2018). This information is conducive for further analysis of the degradation process of specific substrates. In the present study, the physiological characteristics and secretomes of the macro-ascomycete *M. importuna* growing on glucose (G), rice straw (RS), sawdust (SD), wheat grain (WG), and a mixture of equal proportions of the three latter plant materials (MIX) were compared and analyzed, with special emphasis on carbohydrate-active enzymes (CAZYmes). Label-free quantified (LFQ) secreted proteins were identified by nano-liquid chromatography coupled with tandem mass spectrometry (nLC-MS/MS). Concurrently, the activities and gene expression of lignin enzyme, cellulase, xylanase, and amylase during continuous culture were analyzed. This is the first systematic analysis of the secretory proteome of *M. importuna* and will facilitate an understanding of the substrate utilization and nutritional requirements of this fungus.

## Materials and Methods

### Fungal Strain and Culture Conditions

*Morchella importuna* M04 cultivable strain (Strain number: CCTCC AF 2021045) was selected for use in this study as the genome of this strain had recently been sequenced (Liu et al., 2018), and this would be useful for the identification and annotation of functional proteins. The strain was maintained on CYM solid medium (glucose 2%, yeast extract 0.2%, peptone 0.2%, K_2_HPO_4_ 0.1%, MgSO_4_ 0.05%, KH_2_PO_4_ 0.046%, and agar 0.2%).

Five different culture media were used to investigate the mycelium growth rate, biomass, and secretory proteome of *M. importuna* M04. The first, CYM liquid medium (agar-free version of CYM solid medium), was recorded as G. The glucose-free CYM liquid medium containing 2% rice straw powder, 2% sawdust powder, or 2% wheat grain powder described three special plant biomass substrate media that were recorded as RS, SD, and WG, respectively. The final medium, MIX, was composed of equal proportions of rice straw, sawdust, and wheat grain powder (2% in total).

Mycelium growth rate was measured on a 9-cm-diameter plate with the above-mentioned media. Each 0.5-cm-diameter agar block separated from activated mycelia was inoculated in the center of a new 9-cm-diameter CYM plate and cultured at 23°C. Mycelial growth distance within a defined time was determined by the cross-line method. After 14 days, mycelial density and sclerotium morphology were recorded and photographed.

Mycelium biomass was measured by dry weight. Liquid medium (100 ml glucose-free CYM) was placed in a 250-ml conical flask, and the solid carbon sources (RS, SD, WG, and MIX) were weighed, sealed in clear cellophane bags, and placed in the liquid medium, which was then sterilized. Two 0.5-cm-diameter activated agar blocks were inoculated into the sterile medium and were statically cultured at 23°C for 14 days. The resulting mycelium was carefully collected, cleaned with distilled water, dried, and weighed.

Secretory proteome samples were prepared by using the above-mentioned liquid culture conditions and static culture at 23°C for 6 days. After filtration and centrifugation, the supernatant was taken as the crude enzyme solution for subsequent total extracellular protein content determination and secretory protein extraction (Cai et al., 2017).

The preparation of samples for enzyme activity determination was carried out using the same MIX liquid medium and culture conditions as described above. Supernatants were collected at different time points (2, 4, 6, 8, and 10 days post-inoculation, respectively). The mycelium at each corresponding time point was collected, cleaned, and preserved at −80°C for subsequent RNA extraction and quantitative gene expression analysis. Three biological repetitions were performed in each experiment.

### Secreted Protein Preparation

Secreted proteins were extracted using the Tris-phenol method (see Cai et al., 2017, for a detailed protocol). Briefly, triplicate crude enzyme filtrates for each condition were combined equally, and one-fifth volume of Tris-phenol was added, mixed by inversion, and incubated at room temperature for 10 min. Dissolved proteins were collected after centrifugation at 10,000 × *g* for 10 min at 4°C and were precipitated *via* five volumes of cold 0.1 M sodium acetate methanol solution. The precipitated proteins were re-solubilized and denatured in urea solution (9 M urea, 2% CHAPS, 1 mM PMSF, and 50 mM DTT). The Bradford method was employed to determine protein concentration, and a Readyprep 2-D Cleanup kit (Bio-Rad, CA, United States) was used for protein purification. Tryptic (Trypsin Gold, Promega Corp., WI, United States) digestion was conducted overnight at 30°C. The peptides were extracted at 37°C in 100% acetonitrile (ACN) followed by 0.5% trifluoracetic acid, freeze dried, cleaned using C18 ZipTip (Millipore), and reconstituted in 20 μl of 0.1% formic acid and 2% ACN.

### LC-MS/MS Detection and Data Analysis

Peptide samples (20-fold diluted, 2 μl volume) were injected and analyzed using an LTQ-Orbitrap Velos mass spectrometer (Thermo Scientific, MA, United States) coupled with nano-Easy high-performance liquid chromatography equipment (Proxeon, Odense, Denmark). Three technical replicates were run for the LC-MS/MS analysis. The peptides were first trapped onto a 2-cm C18-A1 ASY-Column precolumn (Thermo Scientific, MA, United States) and eluted onto a Biosphere C18 column (75 μm, inner diameter; 15 cm, length; and 3 μm, particle size) *via* a 130-min gradient elution from 0 to 45% buffer B (0.1% formic acid in pure ACN) at a flow rate of 250 nl/min. The LTQ-Orbitrap was set to acquire MS/MS spectra in data-dependent mode as follows: MS survey scans from m/z 300 to 1,600 were collected with a target value of 1,000,000 at a resolution of 30,000 (at m/z 400), and MS/MS spectra were acquired in the linear ion trap with a target value of 10,000 and normalized collision energy of 35%. Precursor ion charge state screening and monoisotopic precursor selection were enabled. Singly charged ions and unassigned charge states were rejected. Dynamic exclusion was enabled with a repeat count of one and an exclusion duration of 30 s.

Raw MS/MS data was searched against the genome of *M. importuna*^1^ by using MaxQuant software (version 3.2.2.0) (Cox and Mann, 2008). The search parameters of MS/MS were set as trypsin digestion, allowing two missed cleavages, carbamidomethylation of cysteine residues was selected as a fixed modification, and the error tolerances on the precursor and fragment ions were 4.5 ppm and 0.5 Da, respectively. For protein identification, the desired false discovery rate at peptide spectrum match and protein was 1%, and at least two unique peptides were required. For abundance calculations, the mass spectrometric signal intensities (LFQ intensities) of peptide precursor ions belonging to each protein were divided by the total abundance of all the detected proteins in each culture condition (Cai et al., 2017). The protein abundance value was calculated from the normalized values of the three technical replicates. Functional annotation and signal peptide data of proteins came from the functional genome of *M. importuna* (Liu et al., 2018).

### Enzyme Activity Assays

Different growth stages of mycelium in MIX liquid medium at 23°C static culture were used to determine dynamic changes in predominant enzyme activities. Enzymatic hydrolyses of the polysaccharides were performed in a sodium acetate buffer solution (SABF, 0.2 M, pH 4.8). The endoglucanase (CMCase), laccase, amylase, xylanase, cellobiohydrolase (pNPCase), and β-glucosidase (pNPGase) activities of the culture supernatants were assayed according to the methods reported by Cai et al. (2017). The endoglucanase (CMCase), xylanase, and amylase activities of the culture supernatants (diluted samples) were assayed using a DNS reagent (10 g 3,5-dinitrosalicylic acid, 20 g sodium hydroxide, 200 g sodium potassium tartrate, 2.0 g redistilled phenol, and 0.50 g sodium sulfite anhydrous per 1,000 ml DNS reagent) against carboxymethyl cellulose sodium salt (CMC-Na), xylan (from beechwood), and soluble starch. CMC-Na, xylan, or starch was dissolved in SABF to a final concentration of 1% (mass/volume percent, m/v %). Then, 0.5 ml of diluted culture supernatants and 1.5 ml CMC-Na, xylan, or starch solution for CMCase, xylanase, or amylase activity assays, respectively, into a 25-ml colorimetric tube. The mixture was mixed gently, and the reaction mixture was incubated for CMCase and xylanase activity measurements at 50°C for 30 min and for amylase activity measurement at 40°C for 10 min. Next, 3 ml of DNS reagent was then added to stop the reaction. A blank tube (with crude enzyme digested by proteinase K, PROMEGA) was used as control to correct any reducing sugar present in the crude enzyme samples. The tubes were placed in boiling water for 10 min, and then 20 ml of distilled water was added. The absorbance was determined at 540 nm. The pNPCase and pNPGase activities were measured by using 4-nitrophenyl β-D-cellobioside (pNPC) and 4-nitrophenyl β-D-glucopyranoside (pNPG) as substrates, respectively. The pNPC or pNPG was dissolved in SABF to a final concentration of 1 mg/ml. Moreover, 50 μl of pNPC solution (containing 1 mg/ml D-glucono-δ-lactone) or 50 μl of pNPG solution and 100 μl of diluted culture supernatants were mixed, and then the mixtures were incubated in 50°C water bath for 30 min. The reaction was stopped by adding 0.15 ml of sodium carbonate solution (10%, m/v). The absorbance was measured at 420 nm. One unit of enzyme activity was defined as the amount of enzyme required to release 1 μmol of glycoside bonds of the substrate per minute under defined assay conditions. The laccase activity was determined by the oxidation rate of 3-ethylbenzothiazole-6-sulfonic acid (ABTS). ABTS was dissolved in SABF to a final concentration of 0.5 mmol. Then, 1 ml of diluted culture supernatants and 2 ml ABTS solution were mixed to start the reaction at 25°C. The absorbance at 420 nm was measured at the beginning and 30 min later. One unit of enzyme activity was defined as the increase of absorbance value caused by the oxidation substrate (ABTS) per minute in 1 ml of enzyme solution under the above-mentioned conditions. Independent triplicate cultures were sampled and analyzed.

### Quantitative Real-Time PCR Analysis

Approximately 100 mg mycelia was ground in liquid nitrogen, and total RNA was extracted using a phenol/SDS method (Zhang et al., 2018). RNA integrity and quantity were checked using an Agilent 2100 Bioanalyzer (Agilent Technologies, CA, United States). The cDNA strand was synthesized using HiScript II Reverse Transcriptase (Vazyme, Nanjing, China) with 500 ng total RNA in a 20-μl reaction according to the manufacturer’s instructions. The primer sequences for the tested genes and the reference gene are listed in Supplementary Table 1. The quantitative RT-PCR was performed using a CFX Connect real-time PCR system (Bio-Rad). Each reaction contained 1 μl each of forward and reverse primer (10 mM), 30 ng sample cDNA, 10 μl AceQ qPCR SYBRMaster Mix (Vazyme), and ddH_2_O to a 20-μl final volume. A two-step RT-PCR protocol was adopted for all amplifications and comprised a denaturation step at 95°C for 2 min, followed by 40 cycles of 95°C for 15 s and 60°C for 20 s, with the fluorescence measured at the end of each cycle. A dissociation curve was generated to verify that a single product was amplified. Transcript levels were normalized with the internal reference gene INIF2 and quantified according to Zhang et al. (2018). Three biological and three technical replicates were analyzed.

## Results

### Physiological Phenotypes of *M. importuna* on Different Media

**FIGURE 1:**
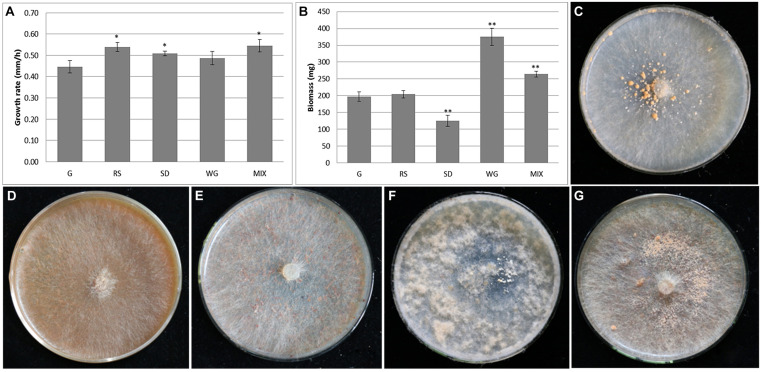
Growth potential of *Morchella importuna* on different media. Mycelium growth rate **(A)** and biomass **(B)** were also measured on the five different media; significant differences are marked by asterisks, *p < 0.05, **p < 0.01. The colony morphology of *M. importuna* was examined on CYM medium (termed G) **(C)**, glucose-free CYM medium containing 2% rice straw powder (RS) **(D)**, 2% sawdust powder (SD) **(E)** or 2% wheat grain powder (WG) **(F)**, and media containing equal proportions of rice straw, sawdust, and wheat grain powders (termed MIX) **(G)**.

The growth potential of *M. importuna* on different plant biomass substrates commonly used in commercial cultivation was compared and analyzed, focusing on mycelial growth rate, colony density, sclerotia, and biomass. The growth rates of *M. importuna* on all four plant biomass media were higher than that of the control G medium (0.45 mm/h). The highest growth rates were recorded on MIX and RS (0.55 and 0.54 mm/h, respectively), and both were significantly different from that of G medium (*p* = 0.014 and *p* = 0.012, respectively), while the growth rates on SD (0.51 mm/h) and WG (0.49 mm/h) were not significantly different frm that of the G control medium (Figure 1A). In contrast to the growth rate, mycelium biomass measurements were highest on WG, followed by MIX, with both significantly higher than that of G (*p* = 1.7e-06 and *p* = 3.6e-05, respectively) (Figure 1B). The mycelium biomass of *M. importuna* on SD medium was significantly lower than that on G (*p* = 8.4e-05), while that on RS was similar to that on G (Figure 1B). On G medium, with glucose as the main carbon source, a small amount of granular sclerotia was evenly distributed around the inoculated block, and creeping aerial hyphae were observed (Figure 1C). The colony morphologies of *M. importuna* on RS and SD media were alike, with sparse and flat aerial hyphae without sclerotia (Figures 1D,E). On WG medium, more aerial hyphae gathered on the surface of the whole medium, and a few sclerotia were occasionally produced around the inoculated block (Figure 1F). On the MIX medium, dense granular sclerotia were produced around the inoculated block (Figure 1G). It was inferred from these growth potential data that *M. importuna* could make best use of WG, followed by MIX, but it was difficult to utilize RS and SD. This was consistent with wheat being an important carbon source in the artificial cultivation of *M. importuna* (Liu et al., 2017). In accordance with the conclusion that *M. crassipes* was found to have a difficulty in decomposing sawdust (Kanwal and Reddy, 2014), *M. importuna* had a weak ability to degrade sawdust as well as rice straw, and this might be related to the extracellular enzyme spectrum of the fungi. In addition, the total extracellular protein content of *M. importuna* grown on each carbon was determined. The most proteins were produced in WG and RS media and followed by SD and MIX media. The extracellular protein content of *M. importuna* in four plant biomass media was extremely higher (*p* < 0.0001) than in G medium (Supplementary Figure 1). It seemed that there was no relationship among the growth rate, mycelium biomass, and the total extracellular protein content.

### Identification and Analysis of the Secretory Proteome of *M. importuna* on Different Media

The secretory proteome of *M. importuna* in the five different media was determined by shotgun mass spectrometry and analyzed. A total of 268 credible proteins were identified (Supplementary Table 2). Protein LFQ intensity was used as the relative quantitative result for the subsequent comparative analysis. Correlation analysis revealed that the correlation coefficient within groups was above 0.98 (Supplementary Table 3), indicating that the data were of sufficient quality for the follow-up comparative analysis. The molecular weights of the identified proteins ranged from 11.8 to 184 kDa, with 90% in the range of 20–120 kDa. The number of secreted proteins identified in the different media was significantly different; 104 proteins were identified in G medium, followed by 108 in RS, 118 in SD, and 127 in MIX, and the largest number of secreted proteins was 215 in WG medium. A comparison of the identified secretory proteins in the three plant biomass media and the control medium (G) revealed that the number of specific proteins in RS (six proteins, 2.3%) and SD (five proteins, 2.0%) was relatively small and was followed by G (six proteins, 2.3%), with WG having the greatest number of specific proteins (77, 27.7%). Only 42 secretory proteins (16.4%) were present in all four media (Supplementary Figure 2). From the total number of secretory proteins and substrate-specific proteins, it was inferred that *M. importuna* produces many proteins that specifically degrade wheat grain components and that the wheat grain components induce a more complex secretome compared with the other tested media.

### Functional Classification of Secretory Proteins of *M. importuna*

**FIGURE 2:**
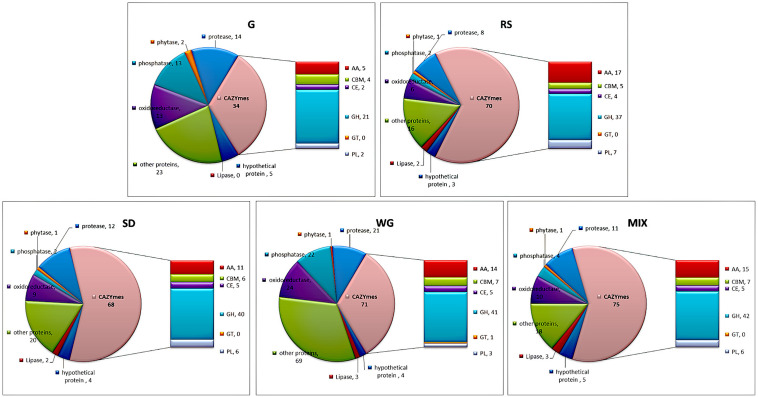
Functional classification of the secretome of *Morchella importuna* in five different culture media. G, CYM medium; RS, glucose-free CYM containing 2% rice straw powder; SD, glucose-free CYM containing 2% sawdust powder; WG, glucose-free CYM containing 2% wheat grain powder; MIX, media containing equal proportions of rice straw, sawdust, and wheat grain powders.

Secretory proteins can be divided into CAZYme, oxidoreductase, lipase, phosphorylase, protease and peptidase, unknown proteins, and other functional proteins. Among these proteins, CAZYme is the predominant protein functional category of environmental biomass degradation, which involves degradation of cellulose, hemicellulose, lignin, pectin, starch, and chitin (Cai et al., 2017). The functional classifications of the secretory proteins of *M. importuna* were different among the five tested media (Figure 2). More CAZYme proteins were identified in plant media (from 68 in SD to 79 in MIX) than in G (34). The CAZYme classes of glycoside hydrolases (GH), auxiliary activities (AA), and polysaccharide lyases (PL) showed the greatest variability across the tested media, with all three of these classes containing the least number of proteins in G medium. In the AA class, except for G, SD had the least proteins (11), followed by WG (14), while RS contained the most AA (17). Like the AA class, the number of PL proteins was highest in RS. Only one glycosyltransferase (GT), another class of CAZYme, was identified in WG, while the other tested media did not contain any GT proteins. The amounts of protease and peptidase proteins were lowest in RS but similar among G, SD, and MIX. Lipases were not identified in G, but two or three lipases were identified in the other four tested media. Many phosphorylated proteins, redox proteins, and other types of proteins were identified in WG. Some proteins were annotated as intracellular proteins, which may be caused by aging and disintegration of the mycelium, including ubiquitin protein, heat shock protein, ribosome size subunit, and tricarboxylate cycle proteins; however, the contents of all these proteins were very low (Supplementary Table 2).

### Comparative Analysis of Secretory Proteins of *M. importuna* in Different Substrates

**FIGURE 3:**
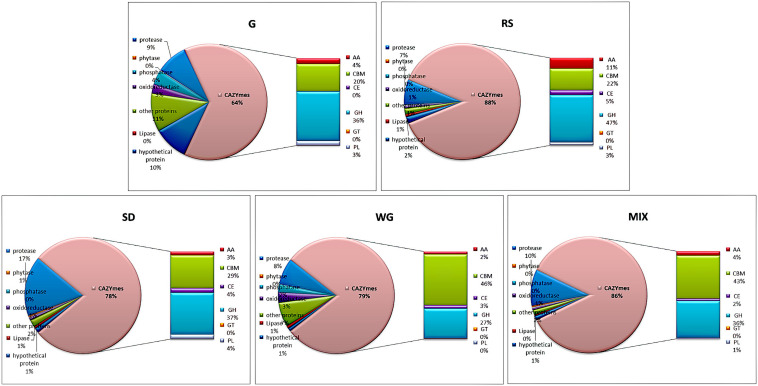
Proportion of the secretome of *Morchella importuna* in five different culture media. G, CYM medium; RS, glucose-free CYM containing 2% rice straw powder; SD, glucose-free CYM containing 2% sawdust powder; WG, glucose-free CYM containing 2% wheat grain powder; MIX, media containing equal proportions of rice straw, sawdust, and wheat grain powders.

The identified secretory proteins were label-free quantified by MaxQuant, and a comparative analysis was conducted by the percentage of LFQ intensity. The protein abundance of CAZYme was the highest in all five media, ranging from 64% in G to 88% in RS and was approximately equal in SD and WG (78 and 79%, respectively) (Figure 3). Protease and peptidase were the next most abundant proteins, followed by other proteins and hydroxy proteins, oxidoreductase, phosphorylase, and lipase (Figure 3).

The abundance of the different classes of CAZYme proteins of *M. importuna* varied in the different culture media. The total protein abundance of GH proteins ranged from 27.17% (WG) to 47.51% (RS), and among these proteins, GH 7 (RS, 20.69%), GH 76 (G, 11.58%), GH 17 (G, 11.67%), GH 35 (SD, 6.98%), and GH 31 (WG, 6.35%) were the most abundant (Supplementary Table 2). CBM35-GH27 (MIM04M26Gene08639) was the main contributor to the high abundance of carbohydrate-binding module (CBM) enzymes, which comprised 44.32% in WG, followed by 39.54, 26.63, and 19.71% in MIX, SD, and RS, respectively, with the lowest abundance detected in G (15.92%). The abundance of AA enzymes was highest in RS with a total amount of 10.88%, while the other four media contained almost equal amounts of this class of enzyme, ranging from 2.37 to 4.11%. The abundance of PL enzymes was higher in G, RS, and SD (ranging from 2.81 to 4.00%) than in WG and MIX (only 0.32 and 0.80%, respectively). The contents of carbohydrate esterase (CE) enzymes were low and almost equal across the five tested media, and a single GT enzyme was only identified in WG with the content comprising only 0.01% (Figure 3).

### Enzyme Activities of *M. importuna* in MIX Medium

**FIGURE 4:**
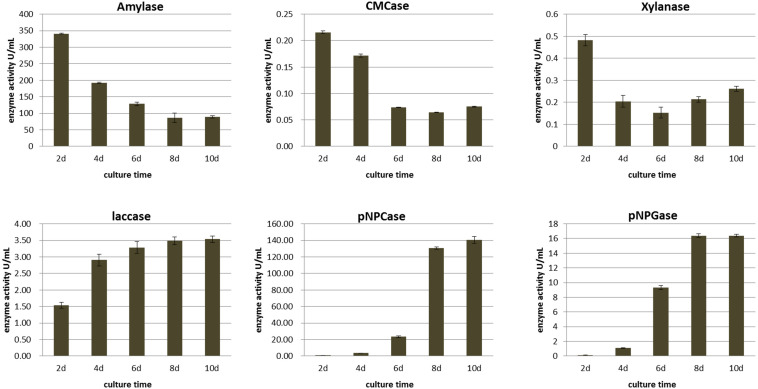
Enzyme activities of *Morchella importuna* on MIX medium.

To explore the dynamic changes of extracellular enzymes in the plant biomass degradation process of *M. importuna*, the activities of enzymes related to starch and lignocellulose degradation of mycelium were measured in MIX medium continuous culture for 10 days. The results are shown in Figure 4.

Amylase activity was the highest at the beginning of the culture period, measuring 340.99 ± 28.16 U/ml and then decreasing rapidly to 86.52 ± 6.84 U/ml at 8 days, with no difference between days 8 and 10 (*p* = 0.5143). The activity of amylase was higher than that of other enzymes, and combined with the data on growth potential in other plant biomass media, it can be inferred that *M. importuna* makes effective use of starch in wheat.

The change trend of cellulase and xylanase activities was consistent, with the highest activity at 2 days (0.215 ± 0.003 and 0.481 ± 0.026 U/ml, respectively), followed by a continuous decrease to the lowest activity at 8 and 6 days, respectively, and then increasing slightly by 10 days, reaching 0.075 ± 0.001 and 0.261 ± 0.011 U/ml, respectively. Laccase activity increased continuously during the whole culture period, but the enzyme activity was weak, with the highest value of 3.538 ± 0.10 U/ml at 10 days. The overall activities of laccase, cellulase, and xylanase were low, which was consistent with the growth potential in specific substrates whereby *M. importuna* was observed to find it difficult to use single rice straw or sawdust as a growth substrate.

The change trend of exocellulase (pNPCase) and β-glucosidase (pNPGase) activities was consistent, with these enzyme activities increasing gradually with continuous culture. During the first 8 days of culture, the enzyme activities increased significantly and reached the highest value at days 10 and 8 for pNPCase (140.48 ± 3.97 U/ml) and pNPGase (16.399 ± 0.186 U/ml), respectively. However, the activity of pNPGase was much lower than that of pNPCase.

### Expression Patterns of Genes Related to Starch and Lignocellulose Degradation of *M. importuna* in MIX Medium

Eight amylase degradation genes were identified in the secretome of *M. importuna* (Tables 1, 2). The expression of MIM04M26Gene03266 (α-1,4-glucose lyase, GH31) increased in the first 6 days of culture and then decreased, while the others showed a downward trend with culture time, which was consistent with the change trend of amylase activity. Amylase-related genes in morel cultivation were previously observed to decrease with cultivation time (Tan et al., 2019). Among the amylase degradation genes examined in this study, the expression level of the MIM04M26Gene03266 gene was the highest at 6 days, followed by MIM04M26Gene04221 (1,4-α-glucan branching enzyme, GH13, CBM20) at 2 days in MIX medium (Table 1).

**TABLE 1:** Expression patterns of the CAZYme genes in MIX medium of *Morchella importuna*.

Gene no.	Gene expression					Annotation classification	CAZYme family
			
	2d	4d	6d	8d	10d		
MIM04M26Gene03003	524.79 ± 33.34	97.38 ± 5.40	11.38 ± 0.54	4.35 ± 0.25	6.51 ± 0.71	Cellulose degradation	GH7,CBM1
MIM04M26Gene05305	1.33 ± 0.08	0.40 ± 0.02	0.73 ± 0.05	0.61 ± 0.04	0.60 ± 0.03	Cellulose degradation	GH6,CBM1
MIM04M26Gene01808	2.78 ± 0.14	0.69 ± 0.26	1.09 ± 0.02	2.07 ± 0.10	0.03 ± 0.01	Cellulose degradation	GH3
MIM04M26Gene02878	0.83 ± 0.19	11.65 ± 0.92	0.61 ± 0.25	0.49 ± 0.20	0.47 ± 0.05	Cellulose degradation	GH3
MIM04M26Gene05691	0.66 ± 0.06	0.77 ± 0.14	0.09 ± 0.02	0.06 ± 0.01	0.01 ± 0.00	Cellulose degradation	GH3
MIM04M26Gene07860	0.17 ± 0.05	0.11 ± 0.03	0.05 ± 0.01	0.02 ± 0.01	0.02 ± 0.01	Cellulose degradation	GH5,CBM46
MIM04M26Gene10806	0.13 ± 0.01	0.18 ± 0.07	0.01 ± 0.00	0.01 ± 0.00	0.03 ± 0.01	Cellulose degradation	AA9,GH61
MIM04M26Gene09935	1.92 ± 0.32	3.26 ± 0.30	1.75 ± 0.12	1.54 ± 0.16	2.10 ± 0.11	Cellulose degradation	GH3
MIM04M26Gene00528	3.64 ± 0.37	2.95 ± 0.77	1.60 ± 0.24	0.08 ± 0.09	0.17 ± 0.02	Cellulose degradation	GH5
MIM04M26Gene06474	62.96 ± 5.68	1.34 ± 0.30	0.56 ± 0.07	0.35 ± 0.03	0.37 ± 0.03	Cellulose degradation	GH6
MIM04M26Gene07177	69.23 ± 6.35	3.34 ± 0.36	0.15 ± 0.09	0.09 ± 0.02	0.14 ± 0.00	Cellulose degradation	AA9,CBM1
MIM04M26Gene06674	9.62 ± 0.77	24.64 ± 1.02	0.52 ± 0.04	0.19 ± 0.04	0.13 ± 0.01	Hemicellulose degradation	GH5
MIM04M26Gene10651	0.21 ± 0.00	0.10 ± 0.01	0.03 ± 0.00	0.04 ± 0.00	0.00 ± 0.00	Hemicellulose degradation	GH26,CBM35
MIM04M26Gene08639	144.46 ± 7.10	43.46 ± 7.54	10.79 ± 1.80	10.23 ± 0.89	7.73 ± 0.24	Hemicellulose degradation	CBM35,GH27
MIM04M26Gene10621	0.99 ± 0.07	1.13 ± 0.07	0.18 ± 0.03	0.29 ± 0.03	0.33 ± 0.02	Hemicellulose degradation	GH10
MIM04M26Gene11386	7.38 ± 0.25	4.73 ± 0.69	6.06 ± 0.25	5.97 ± 0.63	6.52 ± 0.43	Hemicellulose degradation	GH35
MIM04M26Gene03149	3.11 ± 0.26	2.57 ± 1.22	1.90 ± 0.20	2.13 ± 0.15	2.75 ± 0.17	Hemicellulose degradation	GH47
MIM04M26Gene03737	3.15 ± 0.26	9.46 ± 1.19	0.68 ± 0.09	0.28 ± 0.03	0.31 ± 0.04	Hemicellulose degradation	CBM35,GH27
MIM04M26Gene06894	0.76 ± 0.18	0.92 ± 0.24	0.37 ± 0.07	0.37 ± 0.02	0.42 ± 0.10	Hemicellulose degradation	GH35
MIM04M26Gene06324	0.26 ± 0.11	0.32 ± 0.05	0.11 ± 0.02	0.08 ± 0.00	0.09 ± 0.01	Hemicellulose degradation	GH10
MIM04M26Gene07830	4.07 ± 0.39	23.00 ± 1.55	9.52 ± 3.97	3.17 ± 1.60	5.01 ± 0.67	Hemicellulose degradation	GH43,CBM35
MIM04M26Gene10805	40.51 ± 1.25	5.09 ± 0.62	8.93 ± 0.55	8.65 ± 0.34	10.59 ± 1.01	Lignin degradation	AA7
MIM04M26Gene08997	30.58 ± 3.25	25.04 ± 1.47	39.16 ± 2.57	21.59 ± 1.01	33.82 ± 1.94	Lignin degradation	AA3
MIM04M26Gene08278	5.89 ± 0.51	23.88 ± 1.06	1.68 ± 0.12	1.14 ± 0.14	1.03 ± 0.07	Lignin degradation	AA3,AA8,CBM1
MIM04M26Gene01643	0.17 ± 0.04	0.19 ± 0.02	7.04 ± 0.98	1.71 ± 0.21	0.87 ± 0.07	Lignin degradation	AA7
MIM04M26Gene02615	4.34 ± 0.21	11.28 ± 0.71	2.33 ± 0.02	0.89 ± 0.06	0.38 ± 0.04	Lignin degradation	AA7
MIM04M26Gene04737	63.33 ± 4.71	13.25 ± 2.35	10.06 ± 1.61	8.77 ± 1.90	3.33 ± 0.33	Lignin degradation	AA1
MIM04M26Gene10770	0.13 ± 0.02	8.66 ± 0.69	167.07 ± 28.99	357.94 ± 66.16	169.07 ± 12.68	Lignin degradation	AA3
MIM04M26Gene01582	258.54 ± 20.54	9.35 ± 1.73	4.60 ± 0.18	6.28 ± 0.33	7.08 ± 0.66	Lignin degradation	CBM18,AA5
MIM04M26Gene00759	9.69 ± 1.33	25.20 ± 2.41	5.54 ± 1.84	4.82 ± 1.49	3.59 ± 0.26	Pectin degradation	PL1
MIM04M26Gene02058	2.16 ± 0.38	3.38 ± 0.31	0.93 ± 0.13	0.34 ± 0.06	0.15 ± 0.02	Pectin degradation	GH28
MIM04M26Gene02465	0.28 ± 0.04	0.17 ± 0.00	0.10 ± 0.01	0.19 ± 0.01	0.10 ± 0.02	Pectin degradation	PL3
MIM04M26Gene03039	1.41 ± 0.15	5.09 ± 0.17	0.24 ± 0.07	0.20 ± 0.09	0.10 ± 0.03	Pectin degradation	PL3
MIM04M26Gene03491	5.44 ± 0.39	4.91 ± 0.42	1.46 ± 0.08	0.74 ± 0.01	0.92 ± 0.09	Lipase	CE16
MIM04M26Gene06550	6.48 ± 0.94	3.40 ± 0.39	21.06 ± 0.71	33.88 ± 2.65	40.74 ± 4.87	Chitin-binding	GH5
MIM04M26Gene01313	271.18 ± 36.83	68.35 ± 10.70	21.22 ± 2.16	30.20 ± 0.96	32.94 ± 2.67	Chitin-binding	GH17
MIM04M26Gene05979	0.17 ± 0.04	0.15 ± 0.05	2.94 ± 0.18	7.34 ± 0.39	10.90 ± 1.11	Chitin-binding	GH55
MIM04M26Gene08638	21.88 ± 1.76	20.06 ± 1.27	18.89 ± 1.11	6.38 ± 0.27	7.29 ± 0.44	Chitin-binding	CBM18,CE4
MIM04M26Gene00707	17.07 ± 1.74	1.16 ± 0.48	0.69 ± 0.19	0.06 ± 0.01	1.06 ± 0.07	Chitin-binding	CBM18,CE4
MIM04M26Gene09511	22.19 ± 1.62	3.63 ± 0.30	1.93 ± 0.05	2.32 ± 0.09	2.03 ± 0.29	Starch degradation	GH31
MIM04M26Gene04043	7.53 ± 0.66	7.49 ± 0.19	2.10 ± 0.16	0.90 ± 0.10	1.01 ± 0.01	Starch degradation	GH31
MIM04M26Gene03266	0.68 ± 0.14	63.38 ± 3.52	298.97 ± 9.28	49.20 ± 4.23	54.19 ± 4.63	Starch degradation	GH31
MIM04M26Gene04100	12.29 ± 1.24	8.19 ± 0.44	0.27 ± 0.08	0.01 ± 0.01	0.01 ± 0.00	Starch degradation	GH15
MIM04M26Gene04221	184.49 ± 12.24	123.25 ± 17.29	14.49 ± 0.67	13.49 ± 2.55	7.62 ± 0.45	Starch degradation	GH13,CBM20
MIM04M26Gene11143	6.71 ± 0.45	4.78 ± 0.55	0.52 ± 0.04	0.29 ± 0.02	0.32 ± 0.03	Starch degradation	GH13
MIM04M26Gene09512	18.04 ± 1.14	17.75 ± 1.59	1.30 ± 0.22	0.66 ± 0.04	0.26 ± 0.03	Starch degradation	GH13,CBM20
MIM04M26Gene09883	150.65 ± 8.89	26.13 ± 4.21	24.43 ± 0.77	15.84 ± 0.95	15.67 ± 0.95	Starch degradation	GH71
MIM04M26Gene11695	4.31 ± 0.09	4.19 ± 0.72	3.18 ± 0.21	0.85 ± 0.07	1.15 ± 0.14	Starch degradation	GH133

**TABLE 2:** Secretome of *Morchella importuna* in five different substrates.

Protein IDs	G	RS	SD	WG	MIX	CAZY classes	Signal peptide	EC number	*E*-value	Subject annotation
**Cellulose degradation**										
MIM04M26Gene03461	0.00%	0.61%	0.00%	0.01%	0.00%	AA9,CBM1	Y	1.14.99.54	1.48E-108	LPMO
MIM04M26Gene07177	0.58%	0.28%	0.25%	0.24%	0.54%	AA9,CBM1	Y	1.14.99._	1.71E-135	LPMO
MIM04M26Gene10603	0.00%	0.19%	0.00%	0.02%	0.00%	AA9	Y	1.14.99._	5.36E-57	LPMO
MIM04M26Gene10806	0.00%	1.29%	0.12%	0.01%	0.07%	AA9	Y	1.14.99._	9.49E-69	LPMO
MIM04M26Gene01021	0.19%	0.01%	0.00%	0.00%	0.00%	AA9	Y	1.14.99._	6.27E-122	LPMO
MIM04M26Gene06889	0.00%	0.54%	0.00%	0.00%	0.00%	AA9	Y	1.14.99._	7.20E-81	LPMO
MIM04M26Gene04756	0.00%	0.10%	0.03%	0.00%	0.01%	GH3,CBM1	Y	3.2.1.21	0	Beta-glucosidase F
MIM04M26Gene09935	0.39%	0.35%	0.23%	0.26%	0.30%	GH3	N	3.2.1.21	0	Beta-glucosidase A
MIM04M26Gene02878	0.00%	0.30%	0.34%	0.06%	0.13%	GH3	N	3.2.1.21	0	Beta-glucosidase A
MIM04M26Gene05691	0.00%	0.00%	0.15%	0.00%	0.04%	GH3	Y	3.2.1.21	0	Beta-glucosidase
MIM04M26Gene01808	0.00%	0.33%	2.09%	0.00%	0.34%	GH3	Y	3.2.1.21	0	Beta-glucosidase E
MIM04M26Gene00528	0.00%	0.01%	0.00%	0.00%	0.00%	GH5	N	3.2.1.4	0	Endoglucanase
MIM04M26Gene07860	0.00%	0.58%	0.31%	0.08%	0.23%	GH5,CBM46	Y	3.2.1.4	0	Endoglucanase B
MIM04M26Gene04764	0.00%	0.05%	0.09%	0.04%	0.08%	GH5,CBM46	Y	3.2.1.4	0	Endoglucanase B
MIM04M26Gene03526	0.00%	0.25%	0.11%	0.04%	0.12%	GH5,CBM46	Y	3.2.1.4	0	Endoglucanase B
MIM04M26Gene06474	0.73%	1.03%	0.42%	0.27%	0.17%	GH6	Y	3.2.1.91	3.08E-176	Cellobiohydrolase
MIM04M26Gene05305	0.00%	3.31%	0.38%	0.00%	0.20%	GH6,CBM1	Y	3.2.1.91	0	Cellobiohydrolase
MIM04M26Gene03003	0.00%	20.69%	2.95%	0.51%	3.86%	GH7,CBM1	Y	3.2.1.91	0	Reducing end-acting cellobiohydrolase
**Hemicellulose degradation**										
MIM04M26Gene10621	0.03%	4.52%	2.88%	0.92%	4.13%	GH10	Y	3.2.1.8	7.33E-142	Endo-1,4-beta-xylanase
MIM04M26Gene06324	0.08%	0.75%	0.29%	0.07%	0.25%	GH10	Y	3.2.1.8	9.78E-171	Endo-1,4-beta-xylanase
MIM04M26Gene08639	15.93%	19.71%	26.63%	44.32%	39.54%	CBM35,GH27	Y	3.2.1.22	0	Alpha-galactosidase B
MIM04M26Gene03737	0.00%	0.47%	0.59%	0.39%	0.35%	CBM35,GH27	Y	3.2.1.22	0	Alpha-galactosidase D
MIM04M26Gene01233	0.11%	0.13%	0.24%	0.23%	0.40%	CBM35,GH27	Y	3.2.1.49	1.21E-139	Probable alpha-galactosidase B
MIM04M26Gene11386	0.00%	2.23%	6.98%	1.00%	1.85%	GH35	N	3.2.1.23	0	Beta-galactosidase
MIM04M26Gene00587	0.00%	0.29%	0.27%	0.25%	0.13%	GH35	Y	3.2.1.23	0	Beta-galactosidase
MIM04M26Gene06894	0.00%	0.80%	0.69%	0.19%	0.39%	GH35	Y	3.2.1.23	0	Beta-galactosidase A
MIM04M26Gene01326	0.00%	0.18%	0.27%	0.10%	0.14%	GH35	Y		0	Beta-galactosidase
MIM04M26Gene05803	0.00%	0.13%	0.05%	0.03%	0.05%	GH43	Y		6.80E-109	Endo-1,5-alpha-L-arabinosidase
MIM04M26Gene10575	0.00%	0.25%	0.93%	0.00%	0.22%	GH43	Y		4.44E-111	Arabinofuranosidase
MIM04M26Gene07830	0.00%	1.73%	2.42%	0.47%	0.97%	GH43,CBM35	Y		0	Galactan 1,3-beta-galactosidase
MIM04M26Gene11121	0.00%	0.13%	0.00%	0.00%	0.00%	GH26,CBM35	Y	3.2.1.78	5.80E-158	Beta-mannanase
MIM04M26Gene10651	0.00%	1.38%	0.67%	0.14%	0.58%	GH26,CBM35	Y	3.2.1.78	0	Beta-mannanase, partial
MIM04M26Gene03149	0.57%	0.15%	0.36%	0.21%	0.20%	GH47	Y	3.2.1.113	0	Mannosyl-oligosaccharide alpha-1,2-mannosidase
MIM04M26Gene06674	0.00%	0.57%	0.23%	0.44%	0.12%	GH5	Y	3.2.1.78	2.80E-180	Mannan endo-1,4-beta-mannosidase C
MIM04M26Gene00690	0.00%	0.20%	0.44%	0.17%	0.18%	GH51	Y	3.2.1.55	0	Alpha-N-arabinofuranosidase A
MIM04M26Gene05612	0.00%	0.00%	0.00%	0.05%	0.08%	GH51	Y	3.2.1.55	0	Alpha-n-arabinofuranosidase A
MIM04M26Gene05059	0.00%	0.45%	0.50%	0.17%	0.30%	GH95	Y	3.2.1.51	0	Alpha-fucosidase A
MIM04M26Gene01829	0.09%	0.00%	0.00%	0.00%	0.00%	CE1	Y	3.1.1.73	1.23E-72	Ferulic acid esterase (faea)
**Lignin degradation**										
MIM04M26Gene04737	0.00%	0.02%	0.00%	0.07%	0.12%	AA1	Y		0	Laccase-like multicopper oxidase
MIM04M26Gene01497	0.00%	0.00%	0.00%	0.10%	0.00%	AA3	N	1.1.3.13	0	Alcohol oxidase
MIM04M26Gene10770	0.00%	0.00%	0.02%	0.09%	0.07%	AA3	N	1.1.3.13	0	Alcohol oxidase
MIM04M26Gene05928	0.00%	0.01%	0.00%	0.00%	0.00%	AA3	Y		0	Glucose dehydrogenase
MIM04M26Gene08997	0.11%	0.00%	0.00%	0.00%	0.00%	AA3	Y	1.1.99.1	7.29E-97	Glucose-methanol-choline oxidoreductase
MIM04M26Gene06934	0.00%	0.00%	0.00%	0.00%	0.01%	AA3	N	1.1.99.1	0	Glucose-methanol-choline oxidoreductase
MIM04M26Gene08278	0.00%	0.00%	0.23%	0.68%	0.34%	AA3,AA8,CBM1	Y		7.19E-128	Acetyl xylan esterase
MIM04M26Gene01582	2.83%	0.77%	1.20%	0.89%	1.19%	AA5,CBM18	Y	1.2.3.15	0	Glyoxal oxidase
MIM04M26Gene10805	3.17%	1.10%	0.68%	0.66%	1.45%	AA7	Y		1.71E-94	Glucooligosaccharide oxidase
MIM04M26Gene11487	0.00%	0.00%	0.00%	0.05%	0.00%	AA7	N		7.58E-91	6-hydroxy-d-nicotine oxidase
MIM04M26Gene03049	0.00%	0.00%	0.00%	0.00%	0.11%	AA7	Y		6.50E-140	FAD binding domain-containing protein
MIM04M26Gene01643	0.05%	0.19%	0.00%	0.00%	0.00%	AA7	Y		4.45E-153	FAD linked oxidase, N-terminal
MIM04M26Gene02615	0.00%	0.21%	0.00%	0.00%	0.05%	AA7	Y		0	Putative fad binding domain protein
**Pectin degradation**										
MIM04M26Gene03914	0.00%	0.00%	0.33%	0.08%	0.04%	CE12	Y	3.1.1.86	4.89E-101	Rhamnogalacturonan acetylesterase
MIM04M26Gene03520	0.00%	0.06%	0.30%	0.11%	0.19%	CE12	N		1.90E-70	Rhamnogalacturonan acetylesterase rhgt
MIM04M26Gene10611	0.10%	0.00%	0.00%	0.00%	0.00%	GH105	Y		3.56E-125	Predicted protein
MIM04M26Gene08813	0.00%	0.06%	0.34%	0.03%	0.12%	GH105	Y	3.2.1.172	3.68E-144	Cell wall glycosyl hydrolase
MIM04M26Gene03364	0.00%	0.00%	0.00%	0.05%	0.00%	GH28	Y	3.2.1.15	0	Pectin lyase-like protein
MIM04M26Gene02058	1.28%	0.22%	0.03%	0.20%	0.19%	GH28	N		5.16E-147	Coagulation factor 5/8 type domain-containing protein
MIM04M26Gene10507	0.49%	0.00%	0.00%	0.10%	0.11%	GH33,GH93	Y		4.96E-82	Exo-alpha-L-1,5-arabinanase
MIM04M26Gene04876	0.00%	0.00%	0.32%	0.00%	0.08%	GH53	Y		1.44E-116	Arabinogalactan endo-1,4-beta-galactosidase
MIM04M26Gene00759	0.00%	0.66%	0.98%	0.24%	0.28%	PL1	Y	4.2.2.2	3.56E-179	Pectate lyase
MIM04M26Gene05521	0.32%	0.55%	0.36%	0.04%	0.12%	PL1	Y	4.2.2.10	2.44E-157	Putative pectin lyase a precursor protein
MIM04M26Gene10600	0.00%	0.00%	0.00%	0.00%	0.01%	PL1	Y	4.2.2.2	1.96E-114	Pectate lyase A
MIM04M26Gene03039	3.03%	0.02%	0.00%	0.05%	0.00%	PL3	Y	4.2.2.2	2.17E-113	Pectate lyase E
MIM04M26Gene02465	0.00%	1.26%	2.26%	0.00%	0.32%	PL3	Y	4.2.2.2	7.72E-139	Pectate lyase D
MIM04M26Gene03877	0.00%	0.11%	0.04%	0.00%	0.00%	PL4	Y	4.2.2.23	0	Rhamnogalacturonan lyase
MIM04M26Gene09093	0.00%	0.05%	0.09%	0.00%	0.01%	PL4	Y		0	Rhamnogalacturonate lyase
**Starch degradation**										
MIM04M26Gene11143	0.00%	0.03%	0.10%	0.06%	0.19%	GH13	N	3.2.1.1	0	Alpha amylase
MIM04M26Gene09512	0.00%	0.04%	0.92%	0.32%	0.87%	GH13,CBM20	Y	3.2.1.1	0	Alpha-amylase A type-3
MIM04M26Gene04221	0.00%	0.00%	0.00%	0.06%	0.00%	GH13,CBM20	N	2.4.1.18	0	1,4-alpha-glucan-branching enzyme
MIM04M26Gene11695	0.00%	0.00%	0.00%	0.00%	0.00%	GH133	N	3.2.1.33	0	Amylo-alpha-1,6-glucosidase
MIM04M26Gene04100	0.19%	0.00%	0.00%	0.62%	0.28%	GH15	N	3.2.1.3	0	Glucan 1,4-alpha-glucosidase activity
MIM04M26Gene09511	0.45%	0.00%	0.71%	6.36%	5.46%	GH31	Y	3.2.1.20	0	Alpha-1,4-glucan lyase
MIM04M26Gene04043	1.20%	0.97%	1.28%	4.39%	2.76%	GH31	Y	3.2.1.20	0	Alpha-1,4-glucan lyase
MIM04M26Gene03266	1.29%	0.26%	0.59%	1.11%	0.65%	GH31	N	3.2.1.207	0	Alpha-1,4-glucan lyase
MIM04M26Gene09883	2.87%	1.32%	1.46%	0.94%	1.81%	GH71	N	3.2.1.59	2.42E-138	Glucan endo-1,3-alpha-glucosidase
**Chitinase**										
MIM04M26Gene00707	0.00%	0.52%	0.53%	0.12%	0.97%	CE4,CBM18	Y	3.5.1.41	7.46E-123	Chitin deacetylase
MIM04M26Gene08638	1.10%	0.00%	0.00%	0.02%	0.01%	CE4,CBM18	Y		1.10E-103	Chitin binding protein
MIM04M26Gene07866	0.00%	0.00%	0.00%	0.00%	0.06%	CE4,CBM18	Y	3.5.1.41	4.02E-162	Putative chitin deacetylase protein
MIM04M26Gene09204	0.00%	0.00%	0.00%	0.02%	0.02%	GH1	Y	3.2.1.62	0	Beta-glucosidase 34
MIM04M26Gene06550	0.05%	1.49%	1.86%	1.26%	2.16%	GH5	Y		2.07E-177	Glucan 1,3-beta-glucosidase
MIM04M26Gene01313	11.67%	0.75%	0.12%	0.72%	0.32%	GH17	N	3.2.1.58	4.85E-89	Glucan 1,3-beta-glucosidase
MIM04M26Gene00148	0.24%	0.00%	0.14%	0.09%	0.12%	GH20	Y	3.2.1.52	0	*N*-Acetyl-beta-D-glucosaminidase/exochitinase
MIM04M26Gene05979	0.45%	0.66%	3.25%	0.95%	2.02%	GH55	Y		0	Glucan 1,3-beta-glucosidase
MIM04M26Gene04269	0.00%	0.00%	0.00%	0.00%	0.00%	GH72,CBM43	Y		0	Beta-1,3-glucanosyltransferase
MIM04M26Gene02008	11.58%	3.89%	2.39%	4.01%	4.27%	GH76	Y		5.76E-37	Alpha-1,6-mannanase
**Lipase**										
MIM04M26Gene03818	0.00%	0.32%	0.17%	0.10%	0.26%	CE4	Y		0	Polysaccharide deacetylase protein
MIM04M26Gene01797	0.00%	0.28%	0.12%	0.72%	0.29%	CE16	N		9.51E-94	GDSL lipase/acylhydrolase
MIM04M26Gene03491	0.25%	4.29%	3.25%	1.98%	1.29%	CE16	N		4.82E-117	GDSL esterase/lipase
MIM04M26Gene09792	0.00%	0.00%	0.00%	0.48%	0.00%		Y	3.1.1.84	1.98E-95	Carboxylesterase
MIM04M26Gene03055	0.00%	0.39%	0.11%	0.18%	0.13%		Y	3.1.1.8	0	Carboxylesterase
MIM04M26Gene01501	0.00%	0.38%	0.52%	0.13%	0.24%		Y		0	Carboxylesterase
MIM04M26Gene05070	0.00%	0.00%	0.00%	0.00%	0.00%		Y		1.49E-148	Carboxylesterase
										

Thirteen of the 21 cellulose degradation genes in the secretome of *M. importuna* were quantitatively analyzed. The expression patterns of eight genes were similar, showing a steady decrease in expression with the extension of the culture process until 8 days and then slightly increasing at 10 days. These expression patterns were consistent with the changes of CMCase activity (Figure 4). Among the five remaining genes, three (MIM04M26Gene02878, MIM04M26Gene08278, and MIM04M26Gene10806) showed an increase in expression from 2 to 4 days, and then the expression continuously decreased. The expression pattern of the MIM04M26Gene06550 gene was opposite to those of the above-mentioned three genes, first decreasing and then continuously increasing. Among these cellulose-degradation-related genes, the expression level of MIM04M26Gene03003 was the highest at 2 days, followed by MIM04M26Gene07177 (endo-β-1,4-glucanase, AA9) and MIM04M26Gene06474 (cellobiohydrolase, GH6).

Ten of the 21 hemicellulose degradation genes in the secretome were also quantitatively analyzed. Except for MIM04M26Gene08639 (α-galactosidase, GH27), which had a high gene expression, the other nine genes had low levels of expression. MIM04M26Gene11386 (β-galactosidase, GH35) and MIM04M26Gene03149 (mannosyl-oligosaccharide alpha-1,2-mannosidase, GH47) genes were expressed constantly during continuous culture. Expression of MIM04M26Gene03737 (α-galactosidase, GH27) and MIM04M26Gene07830 (galactan 1,3-β-galactosidase, CBM35) genes increased significantly at 4 days and then decreased. The remaining four genes were constantly expressed at 2 and 4 days and then decreased to a stable expression at 6 and 10 days (Table 1).

Among the seven lignin degradation genes, MIM04M26Gene10770 (AA3) had the highest total expression, which continuously increased during the first 8 days of culture, followed by MIM04M26Gene01582 (glyoxal oxidase, AA5/CBM18), which had a high expression at the initial stage of culture and then remained steady over the following 8 days. Two genes, MIM04M26Gene02615 and MIM04M26Gene01643, annotated as AA7 gene family had similar expression patterns, with an initial increase (4 days) followed by a decrease in expression, but the overall expression levels were low. The expression of MIM04M26Gene10805 (glucooligosaccharide oxidase, AA7) and MIM04M26Gene04737 (laccase-like multicopper oxidase, AA1) genes almost continuously decreased, while the expression of MIM04M26Gene08997 (glucose-methanol-choline oxidoreductase, AA3) gene was constant and high throughout the whole culture process.

The expression levels of four detected pectinase genes of *M. importuna* cultured in MIX media were all low, except for MIM04M26Gene02465 (pectate lyase D, PL3) which displayed a continuous decreasing trend during the entire culture process, while the other three genes had an increasing trend in expression at 4 days. Five chitin degradation genes were detected, and four of these showed a decreasing trend in expression, especially MIM04M26Gene01313 (glucan 1,3-β-glucosidase, GH17) and MIM04M26Gene09883 (glucan endo-1,3-α-glucosidase, GH71), which had the highest expression levels at 2 days. In contrast, MIM04M26Gene05979 (GH55), also annotated as glucan 1,3-β-glucosidase, showed a continuous increase in expression throughout the culture process (Table 1).

## Discussion

Fungi secrete extracellular enzymes to degrade biomacromolecules for their own needs. Different fungal species secrete diverse extracellular enzymes in different substrate environments. The CAZYme system is a large repertoire of enzymes secreted by fungi to degrade carbohydrates in the environment, such as cellulose, hemicellulose, lignin, pectin, starch, and chitin (Sista Kameshwar and Qin, 2018). The differences in CAZYme type and quantity are related to species ecological types, such as endophytic, mycorrhizal, pathogenic, and saprophytic (Shah et al., 2009; Jun et al., 2011; Liu et al., 2013; Volke-Sepulveda et al., 2016; Sista Kameshwar and Qin, 2018). A total of 371 CAZYme proteins were predicted in the genome of *M. importuna*, and a comparison with 15 other reference strains revealed that the number of CAZYme genes in *M. importuna* was in the middle among these reference strains, but the number of PL enzymes was significantly higher in *M. importuna* (Liu et al., 2018). *M. importuna* possesses a large number of carbohydrate enzymes involved in the degradation of plant cell walls, which was obviously different from the ectomycorrhizal fungus *Tuber melanosporum* (Liu et al., 2018). However, genomic data cannot provide information about substrate-induced enzyme expression and enzyme activity. Therefore, in this study, the secretory states of *M. importuna* mycelium in five different substrates were analyzed and compared. A total of 89 CAZYme proteins involving the degradation of starch and lignocellulose were identified in the analysis, and their quantity and abundance were significantly different depending on the cultural substrate.

### Amylase

Various amylases can be produced by animals, plants, fungi, and bacteria (Gopinath et al., 2017). Fungal amylase research has predominantly focused on species with industrial applications, such as the saprophytic fungi *Thermomyces* sp., *Aspergillus* sp., *Mucor* sp., *Pycnoporus* sp., and *Saccharomyces* sp. and endophytic fungi like *Preussia minima*, etc., but seldom macro-fungi (Adeniran et al., 2010; Hori et al., 2011; Saranraj, 2013; Zaferanloo et al., 2014). In the process of morel cultivation, most amylopectin and amylose in exogenous nutrition bags were consumed (Tan et al., 2019). Combined with the growth potential and secretome data, *M. importuna* could grow well on the starch-rich medium (Figure 1). Three GH31, three GH13, one GH133, one GH71, and one GH15 secretory proteins were identified in this study. GH31 (MIM04M26Gene09511, MIM04M26Gene04043, and MIM04M26Gene03266) reflected the highest protein abundance in WG and MIX, respectively, suggesting that it could be the key protein of *M. importuna* for starch degradation. The expression of MIM04M26Gene03266 in MIX was the highest at 6 days among all the detected genes (Tables 1, 2). Interestingly, the gene expression levels of MIM04M26Gene09511 and MIM04M26Gene04043 in the continuous culture in MIX media were lower than that of MIM04M26Gene04221 (GH13, CBM20), but the protein abundances were higher in both WG and MIX media, which were probably due to the unequal expression at the protein and gene levels and highlights the importance of protein research. Further exploration is required to determine whether the enzyme distribution and utilization of other macro-ascomycetes is similar to those of morels or not, and investigations should include economic macrofungi with close genetic relationships such as *Verpa* sp., *Helvella* sp., etc. However, it can be concluded that starch-rich grains should be the main metabolite of *M. importuna*.

### Lignin-Degrading Enzymes

LMCOs are phenol-oxidizing enzymes that oxidatively degrade phenol subunits in lignin, improving the accessibility of cellulose and hemicellulose to hydrolytic enzymes in plant litter (Pinto et al., 2012; Reiss et al., 2013; Shirkavand et al., 2016). In addition to lignin degradation, LMCOs also have functions on ion metabolism, parasitism, pigment formation, and fruit body development (Reiss et al., 2013; Madhavan et al., 2014). Lignin degradation is an extracellular process, while the other functions of LMCOs are intracellular. The LMCO genes of morels are responsible for soil extracellular activities related to plant litter decay, and variations in numbers of laccase genes and expression activity were observed in different *Morchella* sp. (Kellner et al., 2007). Some strains of *Morchella* sp. likely possess only one LMCO gene, while some possess two or four to six genes (Kellner et al., 2007). *M. crassipes* had higher laccase activity on wheat grains, wheat bran groundnut shell, and rice straw than on sawdust, apple leaves, carrot peels, orange peels, and pine needles (Kanwal and Reddy, 2014). This was consistent with the observations of the current study, but the overall laccase protein activity was low in this study.

Two different LMCO genes were identified in the artificially cultivated strain SCYDJ1-A1 of *M. importuna*, but only one was actively expressed throughout the growth stages of the morel life cycle (Zhang et al., 2019). Three LMCO genes were identified in the *M. importuna* (M04) genome, all of which belong to AA1 (laccase-like multicopper oxidase family), and two of them were secretory, indicating that they were extracellular proteins (Liu et al., 2018). One of these genes (MIM04M26Gene04737) was identified in the secretome of *M. importuna* in the current study. In the MIX medium, laccase activity increased almost steadily with the extension of culture time. The increases in cellulase and xylanase activities in the later culture stages implied that some cellulose and hemicellulose initially trapped in lignin were released under the action of laccase. It was recently demonstrated that laccase activity in the late stage of metabolism of exogenous nutrient bag was also significantly higher than that in the early stage (Tan et al., 2019). In addition to LMCO, MnP or LiP also plays an important role in lignin degradation. Although the genes encoding these enzymes exist in the genome of *M. importuna*, they were not detected in the secretome in this study. White rot fungi *Pycnoporus cinnabarinus* and *Pycnoporus sanguineus* can produce a single laccase for lignin degradation (Papinutti and Lechner, 2008). However, from the combination of growth rate, biomass, and enzyme activity data, it can be inferred that *M. importuna* might have a limited ability to degrade lignin like the Ascomycetes *Xylaria* (Liers et al., 2006) or *Botryosphaeria* (Dekker et al., 2007). This is in contrast to the white rot fungi in Basidiomycetes, such as *Lentinus edodes* and *Pleurotus ostreatus*, which encode a large number of high-activity laccase genes that effectively degrade and utilize lignin (Fernández-Fueyo et al., 2016; Cai et al., 2017).

### Cellulose-Degrading Enzymes

The complete degradation of cellulose requires the synergism of cellobiose hydrolase (CBHs, EC 3.2.1.91 and EC 3.2.1.176), β-1,4-endoglucanase (EC 3.2.1.4), and β-glucosidase (EC 3.2.1.21) as well as polysaccharide cleavage monooxygenase (LPMO, EC 1.14.99.54, and EC 1.14.99.56) (Coughlan, 1991; Mansfield et al., 1999; Beeson et al., 2015). Cellobiose hydrolases (EC 3.2.1.91 in GH6 and EC 3.2.1.176 in GH7) are the main forces of cellulose degradation by fungi (Morrison et al., 2009; Hori et al., 2013). Three cellobiohydrolases were identified in the secretome of *M. importuna*, with two in GH6 (MIM04M26Gene06474 and MIM04M26Gene05305) and one in GH7 (MIM04M26Gene03003). The abundance of these GH6 and GH7 proteins was higher in RS than in the other media. However, the gene expression of MIM04M26Gene05305 (GH6) on MIX medium was low, which may be related to the specificity induced by RS. The high pNPCase activity detected in MIX medium should be the contribution of GH6 and GH7, especially GH7. Beta-1,4-endoglucanases (EC 3.2.1.4) and LPMOs (EC 1.14.99.54 and EC 1.14.99.56) function for the main chain of cellulose to break down. Four endoglucanases (EC 3.2.1.4) annotated as GH5 were identified. However, the protein abundance of the four GH5 proteins was relatively low. The gene expression of MIM04M26Gene000528 and MIM04M26Gene07860 decreased continuously during culture in MIX (Table 1), consistent with the changing trend of CMCase activity (Figure 4). Five LPMOs (AA9) were also detected, but the protein abundance was low even in RS (Table 2). In terms of further degradation of oligodextran into glucose, β-glucosidases (EC 3.2.1.21) are needed. *Gloeophyllum trabeum*, as a representative of brown rot fungi, can effectively utilize the cellulose of sugarcane bagasse and had a higher abundance of β-glucosidases (GH1 and GH3) for oligosaccharide degradation than that of endoglucanases (GH5 and GH12) (Valadares et al., 2019). Five β-glucosidases (GH3) were identified in the proteomes of *M. importuna*, but their relative protein abundances were low (the highest was 2.09%). Although five β-glucosidase-encoding genes are also expressed at a low level (Tables 1, 2), the *p*NPGase activity was high, especially after 6 days in MIX medium. That is indicating a strongly catalytic ability of β-glucosidases produced by *M. importuna*. In the current study, *M. importuna* is inferred to be weak in degrading cellulose macromolecules into oligomers, and this could be a limiting factor for the utilization of cellulose.

### Hemicellulose-Degrading Enzymes

Hemicellulose, the second most abundant biopolymer present in nature, is a heterogeneous branched polymer including galacto(gluco)-mannan, xylan, and xyloglucan that consists of xylose, mannose, arabinose, glucose, galactose, and sugar acids (Pérez et al., 2002). The heterogeneous nature of xylan means that it requires a variety of enzymes for degradation, including endoxylanases (cleave the β-1,4 glycosidic linkage, GH10, and GH11), galactosidase (release galactose residues, GH27, and GH35), arabinofuranosidases (remove arabinose side-chains, GH43, GH51, and GH53), β-xylosidases (release xylose, GH43, and GH3), acetyl xylan esterases, and feruloyl and ferulic acid esterases (Pérez et al., 2002; Sun et al., 2012). Xylanase acts on the main chain of hemicellulose. Two endo-1,4-β-xylanase (MIM04M26Gene10621, MIM04M26Gene06324, GH10) were identified in the secretome of *M. importuna* in the current study. The former had higher protein abundance and gene expression and might therefore be the key protein for hemicellulose degradation of *M. importuna*. In the degradation of side chains, two L-arabinosidases (GH43), two β-mannanases (GH26/CBM35), two mannosidases (GH47, GH5), two α-*N*-arabinofuranosidase A enzymes (GH51), one α-fucosidase A (GH95), and one ferulic acid esterase (CE1) were detected, but the abundance of these proteins was low (Tables 1, 2).

An α-galactosidase (MIM04M26Gene08639, GH27/CBM35, cleaved galactose residues linked to mannan) was highly abundant in the whole secretome, especially in WG medium where it accounted for 44.32% of the total protein abundance, and this was followed by MIX (39.54%), SD (26.63%), RS (19.71%), and G (15.93%), indicating that this protein had the highest abundance in various medium conditions. The corresponding gene expression was also relatively high in continuous culture in MIX. MIM04M26Gene11386, one of the four β-galactosidases, also had a relatively high protein content, especially in SD (6.98%), which was higher than that in RS (2.23%), MIX (1.85%), and WG (1.00%). Galactosidases are a large class of exoglycoside hydrolases that hydrolyze different substrates linked by terminal α-1, 6-, β-1, 3-, or β-1,6-galactose residues, such as galactose oligomers (melibiose, raffinose, and stachyose), galactomannans, galactoses, and α-D-fucose (Katrolia et al., 2014). In general, the galactosidase content secreted by fungi is low, such as in the secretome of white rot fungus *Lentinula edodes* with microcrystalline cellulase, ligosulfonate, and glucose as substrates and in *Pleurotus ostreatus* with glucose and sawdust as substrates (Cai et al., 2017; Alfaro et al., 2020). Combining the secretome data with the low xylanase activity in MIX (Figure 4), the rate-limiting step of hemicellulose degradation by *M. importuna* is speculated to be the decomposition of lignin skeleton macro-molecules.

### Pectinase

Pectin is a highly hydrated network of polysaccharides rich in galacturonic acid. PLs are the predominant enzymes that degrade these components (Alfaro et al., 2020). Fifteen pectinase degradation proteins were identified in the secretome of *M. importuna*, including two GH105, two GH28, one GH33, one GH53, three PL1, two PL3, two PL4, and two CE12. Thirteen of these were secretory proteins, but the overall abundance was low, and the highest abundance was 3.03% for MIM04M26Gene03039 (PL3) in G and 2.26% for MIM04M26Gene02465 (PL3) in SD. In morel field cultivation, some pectin-degradation-related proteins were identified during the decomposition process in the exogenous nutrition bag (Tan et al., 2019). The solubilization of pectin could improve the accessibility of microorganisms and enzymes, thereby promoting the decomposition of lignocellulose (Shirkavand et al., 2016).

### Lipase and Chitinase

Seven lipases (four carboxylesterases, two GDSL lipases, and one polysaccharide deacetylase) were identified in the secretome of *M. importuna*. Except for two GDSL lipases, the residual exhibits signal peptide (Supplementary Table 2). Five secretory lipases were only detected in plant biomass medium and had low protein abundance. The presence of lipases in the secretome may be induced by a small quantity of lipoproteins in the cell membrane of plant biomass or by the self-dissolution of mycelial cells. Nine proteins related to chitin degradation were identified in the secretome. Seven of them were secretory proteins, and two of the seven had a relatively high abundance. These were MIM04M26Gene01313 (GH17) and MIM04M26Gene02008 (α-1,6-mannanase). The abundance of MIM04M26Gene01313 (GH17) was highest in G (11.67%) and much lower in the other four media (from 0.12% in SD to 0.75% in RS). The same protein with high abundance was also identified in *Penicillium* sp. with glucose as the carbon source (Schneider et al., 2016). The abundance of MIM04M26Gene02008 (α-1,6-mannanase) was higher in all media (11.58% in G *vs*. 2.39% in SD, 3.89% in RS, 4.01% in WG, and 4.27% in MIX, respectively). *Morchella* sp. has the characteristics of rapid developmental aging, and the aging mycelium disintegrates and dies (He et al., 2015a, 2019). The presence of chitinase and the above-mentioned lipases may be responsible for the decomposition and utilization of aged mycelia cells during culture. In morel cultivation, stored lipids in the mycelial network and sclerotia cells in soil are an important energy source for late sexual reproduction (Liu et al., 2017; He et al., 2018). The existence of secretory lipase and chitinase may imply that the lipid in mycelium cells is absorbed and utilized after decomposition *in vitro* rather than being transported and stored for sexual reproduction as during *in vivo* decomposition. In the late growth period, the mycelium cells in the soil have been in an inactive state of aging, and the re-decomposition and re-utilization of aging mycelium is therefore economically effective.

In conclusion, the physiological characteristics and secretomes of *M. importuna* growing on glucose and three plant biomasses commonly used in artificial cultivation were compared and analyzed in this paper. The most suitable medium for *M. importuna* was wheat-rich medium, followed by glucose, rice straw, and sawdust. The combined data of growth potential, secretome, extracellular enzyme activity, and gene expression on different substrates led us to infer that *M. importuna* was weak in lignocellulose degradation but a good starch decomposer. Specifically, the ability to degrade cellulose into oligosaccharides was weaker compared with further degradation into monosaccharides. In addition, *M. importuna* had a strong ability to decompose all kinds of hemicellulose glycosidic bonds but was weak in lignin degradation. Furthermore, the presence of lipase and chitinase implied that *M. importuna* was capable of decomposing its own mycelia *in vitro*, which might be related to cell wall decomposing and morphogenesis during mycelia growth. This study promotes an understanding of the nutritional metabolism and substrate requirements of *M. importuna* and may have implications for the artificial cultivation of commercially important fungi.

## Data Availability Statement

The datasets presented in this study can be found in online repositories. The names of the repository/repositories and accession number(s) can be found in the article/Supplementary Material.

## Author Contributions

WL, YC, and WH conceived and designed the experiments. XM, YC, QQZ, QZ, and JS performed the experiments. WL, FY, QZ, and YC analyzed the data. XM, FY, and WL provided the funding support. WL and YC wrote the manuscript. All authors contributed to the article and approved the submitted version.

## Conflict of Interest

The authors declare that the research was conducted in the absence of any commercial or financial relationships that could be construed as a potential conflict of interest.

## Footnotes

1 https://www.ncbi.nlm.nih.gov/nuccore/QOKS00000000.1/

## Supplementary Material

The Supplementary Material for this article can be found online at: https://www.frontiersin.org/articles/10.3389/fmicb.20 ± 31.636344/full#supplementary-material

## References

[B1] Adeniran H. A. Abiose S. H. Ogunsua A. O. (2010). Production of fungal β-amylase and amyloglucosidase on some nigerian agricultural residues. *Food Bioprocess Technol.* 3 693–698. 10.1007/s11947-008-0141-3

[B2] Alfaro M. Majcherczyk A. Kües U. Ramírez L. Pisabarro A. G. (2020). Glucose counteracts wood-dependent induction of lignocellulolytic enzyme secretion in monokaryon and dikaryon submerged cultures of the white-rot basidiomycete *Pleurotus ostreatus*. *Sci. Rep.* 10:12421. 10.1038/s41598-020-68969-1 32709970 PMC7381666

[B3] Beeson W. T. Vu V. V. Span E. A. Phillips C. M. Marletta M. A. (2015). Cellulose degradation by polysaccharide monooxygenases. *Annu. Rev. Biochem.* 84 923–946. 10.1146/annurev-biochem-060614-034439 25784051

[B4] Bouws H. Wattenberg A. Zorn H. (2008). Fungal secretomes—nature’s toolbox for white biotechnology. *Appl. Microbiol. Biotechnol.* 80:381. 10.1007/s00253-008-1572-5 18636256

[B5] Buscot F. Kottke I. (1990). The association of *Morchella rotunda* (Pers.) Boudier with roots of *Picea abies* (L.) Karst. *New Phytol.* 116 425–430. 10.1111/j.1469-8137.1990.tb00528.x 33874101

[B6] Buscot F. Roux J. (1987). Association between living roots and ascocarps of *Morchella rotunda*. *Trans. Br. Mycol. Soc.* 89 249–252. 10.1016/S0007-1536(87)80162-6

[B7] Cai Y. L. Gong Y. H. Liu W. Hu Y. Chen L. F. Yan L. L. (2017). Comparative secretomic analysis of lignocellulose degradation by *Lentinula edodes* grown on microcrystalline cellulose, lignosulfonate and glucose. *J. Proteom.* 163 92–101. 10.1016/j.jprot.2017.04.023 28483534

[B8] Cavazzoni V. Manzoni M. (1994). Extracellular cellulolytic complex from *Morchella conica*: production and purification. *LWT Food Sci. Technol.* 27 73–77. 10.1006/fstl.1994.1015

[B9] Coughlan M. P. (1991). Mechanisms of cellulose degradation by fungi and bacteria. *Anim. Feed Sci. Technol.* 32 77–100. 10.1016/0377-8401(91)90012-H

[B10] Cox J. Mann M. (2008). MaxQuant enables high peptide identification rates, individualized p.p.b.-range mass accuracies and proteome-wide protein quantification. *Nat. Biotechnol.* 26 1367–1372. 10.1038/nbt.1511 19029910

[B11] Dekker R. F. Barbosa A. M. Giese E. C. Godoy S. D. Covizzi L. G. (2007). Influence of nutrients on enhancing laccase production by *Botryosphaeria rhodina* MAMB-05. *Int. Microbiol.* 10 177–185. 18075999

[B12] Fan L. Soccol C. R. Pandey A. (2008). “Mushroom production,” in *Current Developments in Solid-state Fermentation*, eds Pandey A. Soccol C. R. Larroche C. (New York, NY: Springer New York), 253–274.

[B13] Fernández-Fueyo E. Ruiz-Dueñas F. J. López-Lucendo M. F. Pérez-Boada M. Rencoret J. Gutiérrez A. (2016). A secretomic view of woody and nonwoody lignocellulose degradation by *Pleurotus ostreatus*. *Biotechnol. Biofuels* 9:49. 10.1186/s13068-016-0462-9 26933449 PMC4772462

[B14] Girard V. Dieryckx C. Job C. Job D. (2013). Secretomes: the fungal strike force. *Proteomics* 13 597–608. 10.1002/pmic.201200282 23349114

[B15] Gopinath S. C. B. Anbu P. Arshad M. K. M. Lakshmipriya T. Voon C. H. Hashim U. (2017). Biotechnological processes in microbial amylase production. *BioMed Res. Int.* 2017:1272193. 10.1155/2017/1272193 28280725 PMC5322433

[B16] Gramss G. Günther T. Fritsche W. (1998). Spot tests for oxidative enzymes in ectomycorrhizal, wood-, and litter decaying fungi. *Mycol. Res.* 102 67–72. 10.1017/S095375629700436X

[B17] He P. Wang K. Cai Y. Hu X. Zheng Y. Zhang J. (2018). Involvement of autophagy and apoptosis and lipid accumulation in sclerotial morphogenesis of *Morchella importuna*. *Micron* 109 34–40. 10.1016/j.micron.2018.03.005 29614428

[B18] He P. X. Cai Y. L. Liu S. M. Han L. Huang L. N. Liu W. (2015a). Morphological and ultrastructural examination of senescence in *Morchella elata*. *Micron* 78:6. 10.1016/j.micron.2015.07.010 26281756

[B19] He P. X. Liu W. Cai Y. L. He X. S. (2015b). Strain identification and phylogenetic analysis of cultivated and wild strains of Morchella belonging to elata clade in China. *J. Zhengzhou Univ. Light Ind.* 30 26–29.

[B20] He P. X. Yu M. Cai Y. L. Liu W. Wang W. S. Wang S. H. (2019). Effect of ageing on culture and cultivation of the culinary-medicinal mushrooms, Morchella importuna and M. sextelata (Ascomycetes). *Int. J. Med. Mushrooms* 21 1089–1098. 10.1615/IntJMedMushrooms.2019032891 32450018

[B21] Hervey A. Bistis G. Leong I. (1978). Cultural studies of single ascospore isolates of *Morchella esculenta*. *Mycologia* 70 1269–1274. 10.2307/3759331

[B22] Hori C. Gaskell J. Igarashi K. Samejima M. Hibbett D. Henrissat B. (2013). Genomewide analysis of polysaccharides degrading enzymes in 11 white- and brown-rot Polyporales provides insight into mechanisms of wood decay. *Mycologia* 105 1412–1427. 10.3852/13-072 23935027

[B23] Hori C. Igarashi K. Katayama A. Samejima M. (2011). Effects of xylan and starch on secretome of the basidiomycete Phanerochaete chrysosporium grown on cellulose. *FEMS Microbiol. Lett.* 321 14–23. 10.1111/j.1574-6968.2011.02307.x 21569082

[B24] Jun H. Kieselbach T. Jönsson L. J. (2011). Enzyme production by filamentous fungi: analysis of the secretome of *Trichoderma reesei* grown on unconventional carbon source. *Microb. Cell Fact.* 10:68. 10.1186/1475-2859-10-68 21861877 PMC3179704

[B25] Kanwal H. K. Reddy M. S. (2011). Effect of carbon, nitrogen sources and inducers on ligninolytic enzyme production by *Morchella crassipes*. *World J. Microbiol. Biotechnol.* 27 687–691. 10.1007/s11274-010-0507-3

[B26] Kanwal H. K. Reddy M. S. (2014). Influence of sclerotia formation on ligninolytic enzyme production in *Morchella crassipes*. *J. Basic Microbiol.* 54 S63–S69. 10.1002/jobm.201200802 23712903

[B27] Katrolia P. Rajashekhara E. Yan Q. Jiang Z. (2014). Biotechnological potential of microbial α-galactosidases. *Crit. Rev. Biotechnol.* 34 307–317. 10.3109/07388551.2013.794124 23937250

[B28] Kellner H. Luis P. Buscot F. (2007). Diversity of laccase-like multicopper oxidase genes in Morchellaceae: identification of genes potentially involved in extracellular activities related to plant litter decay. *FEMS Microbiol. Ecol.* 61 153–163. 10.1111/j.1574-6941.2007.00322.x 17466024

[B29] Liers C. Ullrich R. Steffen K. T. Hatakka A. Hofrichter M. (2006). Mineralization of 14C-labelled synthetic lignin and extracellular enzyme activities of the wood-colonizing ascomycetes *Xylaria hypoxylon* and *Xylaria polymorpha*. *Appl. Microbiol. Biotechnol.* 69 573–579. 10.1007/s00253-005-0010-1 16021487

[B30] Liu G. Zhang L. Wei X. Zou G. Qin Y. Ma L. (2013). Genomic and secretomic analyses reveal unique features of the lignocellulolytic enzyme system of *Penicillium decumbens*. *PLoS One* 8:e55185. 10.1371/journal.pone.0055185 23383313 PMC3562324

[B31] Liu W. Chen L. F. Cai Y. L. Zhang Q. Q. Bian Y. B. (2018). Opposite polarity monospore genome de novo sequencing and comparative analysis reveal the possible heterothallic life cycle of *Morchella importuna*. *Int. J. Mol. Sci.* 19:2525. 10.3390/ijms19092525 30149649 PMC6164635

[B32] Liu W. Zhang Y. He P. X. (2017). *Morel Biology and Cultivation.* Changchun: Jilin science and Technology Press.

[B33] Madhavan S. Krause K. Jung E.-M. Kothe E. (2014). Differential regulation of multi-copper oxidases in *Schizophyllum commune* during sexual development. *Mycol. Prog.* 13:1009. 10.1007/s11557-014-1009-8

[B34] Mansfield S. D. Mooney C. Saddler J. N. (1999). Substrate and enzyme characteristics that limit cellulose hydrolysis. *Biotechnol. Prog.* 15 804–816. 10.1021/bp9900864 10514250

[B35] McCotter S. W. Horianopoulos L. C. Kronstad J. W. (2016). Regulation of the fungal secretome. *Curr. Genet.* 62 533–545. 10.1007/s00294-016-0578-2 26879194

[B36] Morrison M. Pope P. B. Denman S. E. McSweeney C. S. (2009). Plant biomass degradation by gut microbiomes: more of the same or something new? *Curr. Opin. Biotechnol.* 20 358–363. 10.1016/j.copbio.2009.05.004 19515552

[B37] Ower R. D. Mills G. L. Malachowski J. A. (1986). *Cultivation of Morchella. US4866878A.*

[B38] Papinutti L. Lechner B. (2008). Influence of the carbon source on the growth and lignocellulolytic enzyme production by *Morchella esculenta* strains. *J. Ind. Microbiol. Biotechnol.* 35 1715–1721. 10.1007/s10295-008-0464-0 18791758

[B39] Pérez J. Muñoz-Dorado J. de la Rubia T. Martínez J. (2002). Biodegradation and biological treatments of cellulose, hemicellulose and lignin: an overview. *Int. Microbiol.* 5 53–63. 10.1007/s10123-002-0062-3 12180781

[B40] Phanpadith P. Yu Z. Yu D. Phongsavath S. Shen K. Zheng W. (2020). Promotion of maize growth by a yellow morel, *Morchella crassipes*. *Symbiosis* 80 33–41. 10.1007/s13199-019-00651-1

[B41] Pinto P. A. Dias A. A. Fraga I. Marques G. Rodrigues M. A. M. Colaço J. (2012). Influence of ligninolytic enzymes on straw saccharification during fungal pretreatment. *Bioresour. Technol.* 111 261–267. 10.1016/j.biortech.2012.02.068 22406100

[B42] Reiss R. Ihssen J. Richter M. Eichhorn E. Schilling B. Thöny-Meyer L. (2013). *Laccase versus* laccase-like multi-copper oxidase: a comparative study of similar enzymes with diverse substrate spectra. *PLoS One* 8:e65633. 10.1371/journal.pone.0065633 23755261 PMC3670849

[B43] Robbins W. J. Hervey A. (1959). Wood extract and growth of *Morchella*. *Mycologia* 51 356–363. 10.1080/00275514.1959.12024823

[B44] Saranraj P. (2013). Fungal amylase – a review. *Int. J. Microbiol. Res.* 4 203–211. 10.5829/idosi.ijmr.2013.4.2.75170

[B45] Schneider W. D. H. Gonçalves T. A. Uchima C. A. Couger M. B. Prade R. Squina F. M. (2016). *Penicillium echinulatum* secretome analysis reveals the fungi potential for degradation of lignocellulosic biomass. *Biotechnol. Biofuels* 9:66. 10.1186/s13068-016-0476-3 26989443 PMC4794826

[B46] Shah P. Atwood J. A. Orlando R. El Mubarek H. Podila G. K. Davis M. R. (2009). Comparative proteomic analysis of *Botrytis cinerea* secretome. *J. Proteome Res.* 8 1123–1130. 10.1021/pr8003002 19140674

[B47] Shirkavand E. Baroutian S. Gapes D. J. Young B. R. (2016). Combination of fungal and physicochemical processes for lignocellulosic biomass pretreatment – a review. *Renew. Sustain. Energy Rev.* 54 217–234. 10.1016/j.rser.2015.10.003

[B48] Sista Kameshwar A. K. Qin W. (2018). Comparative study of genome-wide plant biomass-degrading CAZymes in white rot, brown rot and soft rot fungi. *Mycology* 9 93–105. 10.1080/21501203.2017.1419296 30123665 PMC6059041

[B49] Stark C. Babik W. Durka W. (2009). Fungi from the roots of the common terrestrial orchid Gymnadenia conopsea. *Mycol. Res.* 113 952–959. 10.1016/j.mycres.2009.05.002 19486943

[B50] Sun J. Tian C. Diamond S. Glass N. L. (2012). Deciphering transcriptional regulatory mechanisms associated with hemicellulose degradation in *Neurospora crassa*. *Eukaryotic Cell* 11 482–493. 10.1128/ec.05327-11 22345350 PMC3318299

[B51] Tan H. Kohler A. Miao R. Liu T. Zhang Q. Zhang B. (2019). Multi−omic analyses of exogenous nutrient bag decomposition by the black morel *Morchella importuna* reveal sustained carbon acquisition and transferring. *Environ. Microbiol.* 21 3909–3926. 10.1111/1462-2920.14741 31314937

[B52] Tjalsma H. Bolhuis A. Jongbloed J. D. H. Bron S. van Dijl J. M. (2000). Signal peptide-dependent protein transport in *Bacillus subtilis*: a genome-based survey of the secretome. *Microbiol. Mol. Biol. Rev.* 64 515–547. 10.1128/mmbr.64.3.515-547.2000 10974125 PMC99003

[B53] Valadares F. Gonçalves T. A. Damasio A. Milagres A. M. F. Squina F. M. Segato F. (2019). The secretome of two representative lignocellulose-decay basidiomycetes growing on sugarcane bagasse solid-state cultures. *Enzyme Microb. Technol.* 130:109370. 10.1016/j.enzmictec.2019.109370 31421724

[B54] Volke-Sepulveda T. Salgado-Bautista D. Bergmann C. Wells L. Gutierrez-Sanchez G. Favela-Torres E. (2016). Secretomic insight into glucose metabolism of *Aspergillus brasiliensis* in solid-state fermentation. *J. Proteome Res.* 15 3856–3871. 10.1021/acs.jproteome.6b00663 27548361

[B55] Yu D. Bu F. F. Hou J. J. Kang Y. X. Yu Z. D. (2016). A morel improved growth and suppressed *Fusarium* infection in sweet corn. *World J. Microbiol. Biotechnol.* 32:192. 10.1007/s11274-016-2151-z 27718147

[B56] Zaferanloo B. Bhattacharjee S. Ghorbani M. M. Mahon P. J. Palombo E. A. (2014). Amylase production by *Preussia minima*, a fungus of endophytic origin: optimization of fermentation conditions and analysis of fungal secretome by LC-MS. *BMC Microbiol.* 14:55. 10.1186/1471-2180-14-55 24602289 PMC3995912

[B57] Zhang Q. Miao R. Liu T. Huang Z. Peng W. Gan B. (2019). Biochemical characterization of a key laccase-like multicopper oxidase of artificially cultivable *Morchella importuna* provides insights into plant-litter decomposition. *3 Biotech* 9:171. 10.1007/s13205-019-1688-6 30997308 PMC6456629

[B58] Zhang Q. Q. Liu W. Cai Y. L. Lan A. F. Bian Y. B. (2018). Validation of internal control genes for quantitative real-time PCR gene expression analysis in *Morchella*. *Molecules* 23:2331. 10.3390/molecules23092331 30213125 PMC6225436

