# Supplementary figures and images for: Physiological Characteristics and Comparative Secretome Analysis of Morchella importuna Grown on Glucose, Rice Straw, Sawdust, Wheat Grain, and MIX Substrates
Source: Front Microbiol. 2021 May 25;12:636344. doi: 10.3389/fmicb.2021.636344 (PMC8185036; doi:10.3389/fmicb.2021.636344)

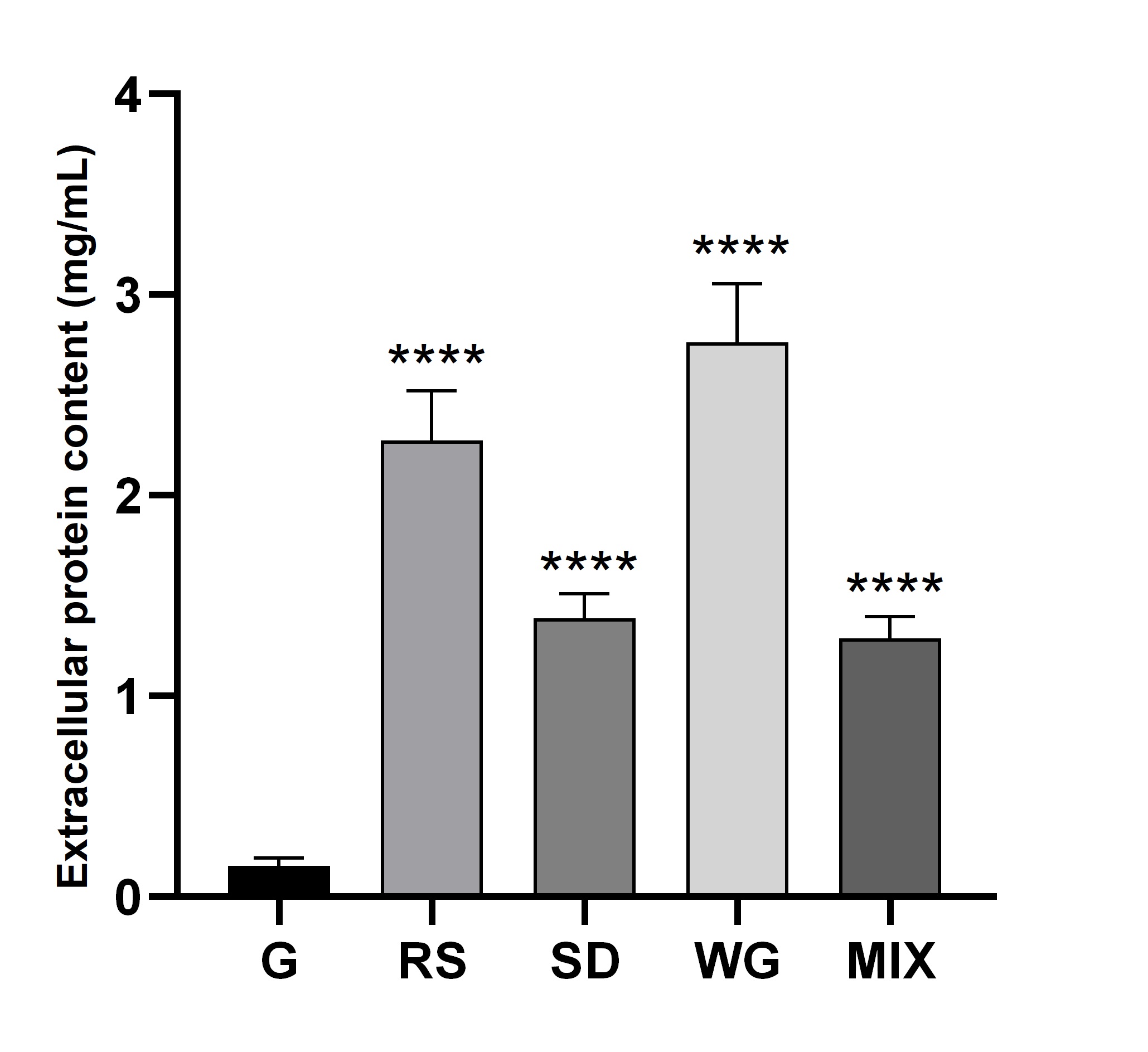

Supplement: Supplementary file 1 [file Image_1.JPEG]

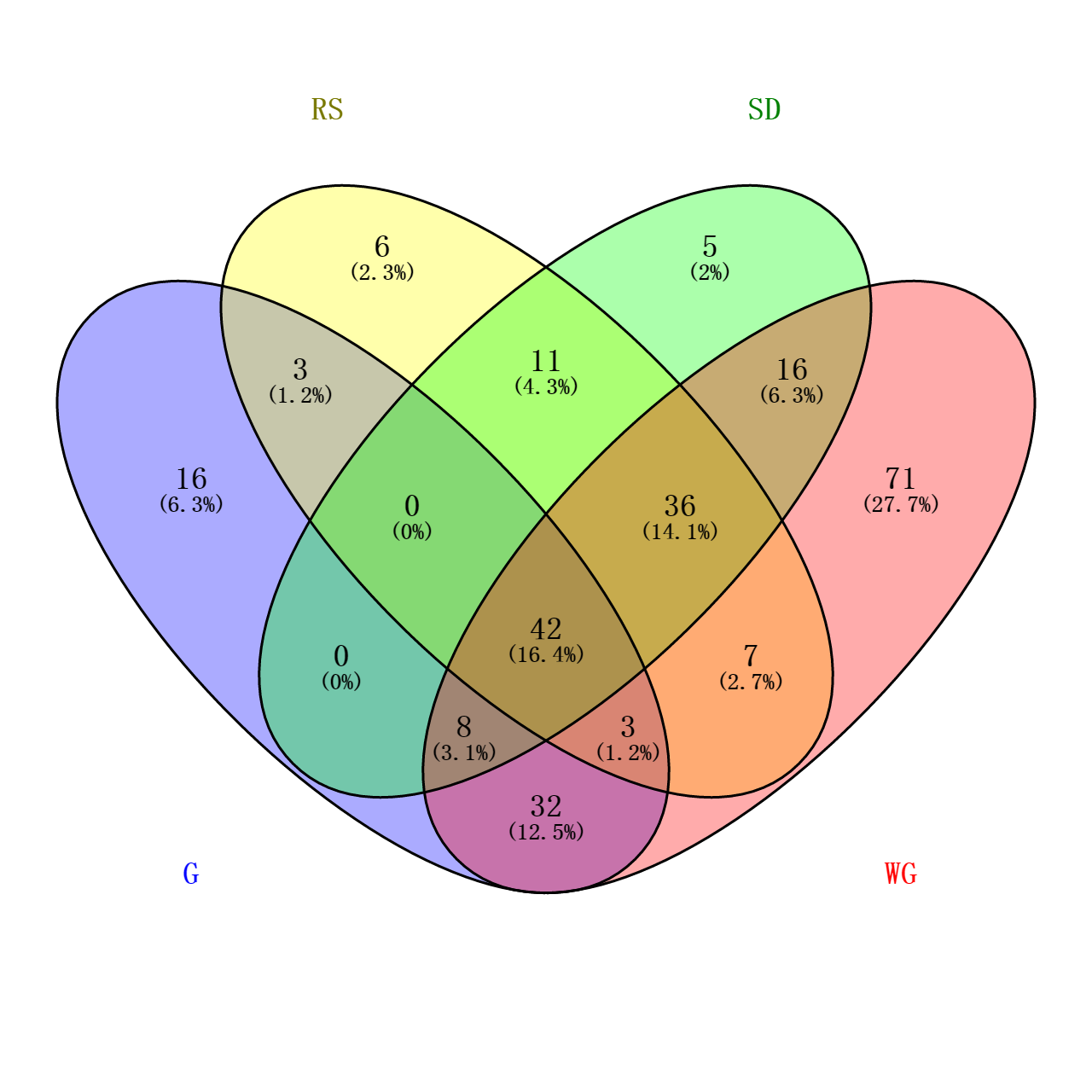

Supplement: Supplementary file 2 [file Image_2.PNG]
